# Structural response of G protein binding to the cyclodepsipeptide inhibitor FR900359 probed by NMR spectroscopy†

**DOI:** 10.1039/d4sc01950d

**Published:** 2024-07-04

**Authors:** Christian Bonifer, Wiebke Hanke, Jonas Mühle, Frank Löhr, Johanna Becker-Baldus, Jessica Nagel, Gebhard F. X. Schertler, Christa E. Müller, Gabriele M. König, Daniel Hilger, Clemens Glaubitz

**Affiliations:** a Institute of Biophysical Chemistry, Centre of Biomolecular Magnetic Resonance, Goethe University Frankfurt Max-von-Laue Str. 9 60438 Frankfurt Germany glaubitz@em.uni-frankfurt.de; b Institute for Pharmaceutical Biology, University of Bonn Nussallee 6 53115 Bonn Germany; c Division of Biology and Chemistry, Laboratory of Biomolecular Research, Paul Scherrer Institute Forschungsstr. 111, 5232 Villigen PSI Switzerland; d Department of Pharmaceutical & Medicinal Chemistry, Pharmaceutical Institute, University of Bonn An der Immenburg 4 53121 Bonn Germany; e Department of Pharmaceutical Chemistry, University of Marburg 35037 Marburg Germany daniel.hilger@pharmazie.uni-marburg.de

## Abstract

The cyclodepsipeptide FR900359 (FR) and its analogs are able to selectively inhibit the class of G_q_ proteins by blocking GDP/GTP exchange. The inhibitor binding site of G_q_ has been characterized by X-ray crystallography, and various binding and functional studies have determined binding kinetics and mode of inhibition. Here we investigate isotope-labeled FR bound to the membrane-anchored G protein heterotrimer by solid-state nuclear magnetic resonance (ssNMR) and in solution by liquid-state NMR. The resulting data allowed us to identify regions of the inhibitor which show especially pronounced effects upon binding and revealed a generally rigid binding mode in the *cis* conformation under native-like conditions. The inclusion of the membrane environment allowed us to show a deep penetration of FR into the lipid bilayer illustrating a possible access mode of FR into the cell. Dynamic nuclear polarization (DNP)-enhanced ssNMR was used to observe the structural response of specific segments of the Gα subunit to inhibitor binding. This revealed rigidification of the switch I binding site and an allosteric response in the α5 helix as well as suppression of structural changes induced by nucleotide exchange due to inhibition by FR. Our NMR studies of the FR-G protein complex conducted directly within a native membrane environment provide important insights into the inhibitors access *via* the lipid membrane, binding mode, and structural allosteric effects.

## Abstract 2

The selective G_q_ inhibitor FR900359 was probed by NMR spectroscopy bound to the membrane-anchored G_q_ heterotrimer, revealing its mode of membrane interaction, protein binding and allosteric protein response.

## Graphical abstract

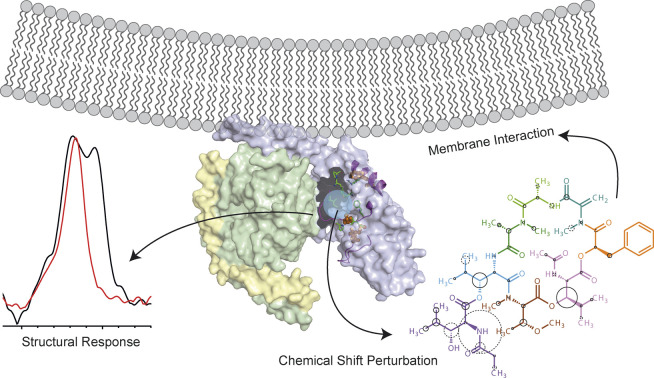

## Introduction

**Fig. 1:**
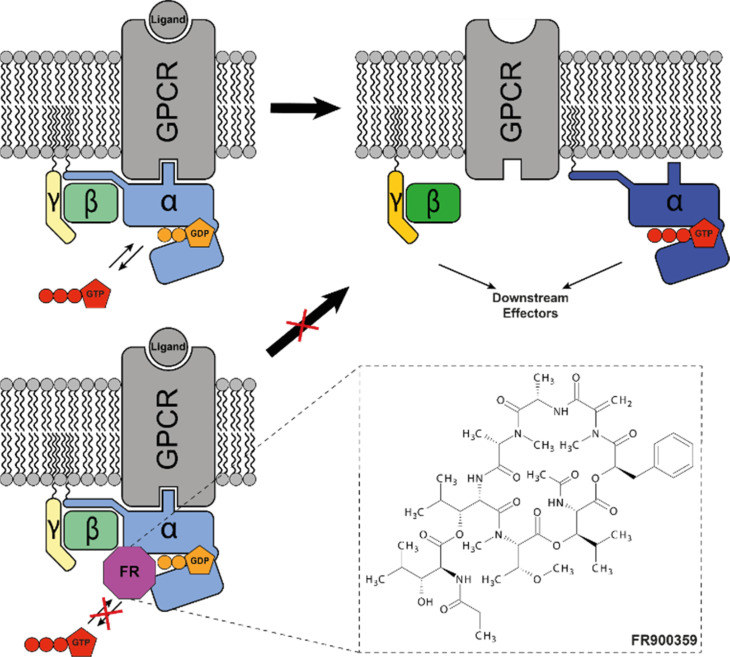
General model of G_q_ protein function and inhibition by FR. Extracellular ligands bind to membrane-embedded GPCRs, which conduct the signal to the heterotrimeric G protein. The latter is lipid-anchored to the membrane and the Gα subunit is GDP bound in its inactive state. Upon ligand-induced conformational changes of the GPCR, the G protein undergoes nucleotide exchange of GDP for GTP and the subunits dissociate. Gα and Gβγ are now able to conduct the signal to other downstream effectors in the signaling pathway (top). Once GTP is hydrolyzed by the Gα subunit, the system reverts back to its inactive state (not shown). In the case of FR-inhibition, the selective binding of the cyclodepsipeptide to Gα_q_ proteins blocks the nucleotide exchange and thus keeps the G protein in its inactive heterotrimeric state, prohibiting signal conduction (bottom).

The protein superfamily of G protein-coupled receptors (GPCRs) is involved in a plethora of cellular signaling pathways across different organisms. Those integral membrane proteins are activated by extracellular ligands, which induce a conformational change in the receptor upon binding, leading to the transduction of this extracellular signal to the intracellular side, where the eponymous G protein is located.^1^ G proteins are membrane-anchored heterotrimers of the Gα, Gβ and Gγ subunit, of which the Gα subunit forms the main contact site with the GPCR. Gα consists of an alpha-helical domain and a GTP-binding domain that acts as a GTPase by binding and hydrolyzing the nucleotide guanosine triphosphate (GTP).^2^ In the inactive state, Gα is bound to guanosine diphosphate (GDP) and Gβγ. Upon GPCR activation, the conformational changes of the receptor lead to a release of GDP from Gα and binding of GTP, which is readily available inside the cell in a concentration surplus over GDP of 10 : 1.^3^ The GTP-bound active state of Gα dissociates from the Gβγ domains and the GPCR to interact with downstream effectors of the activated signaling pathway, leading to further cellular responses to the external ligand (Fig. 1, top). Once the bound GTP is hydrolyzed, the G protein reverts back into its inactive form and the heterotrimer can bind the GPCR again.^4^ This is the general model of signal transduction *via* GPCRs and a large number of those receptors have evolved to accommodate a likewise large number of different ligands and activators. But this wide range of receptors is coupled to only a relatively small number of G protein families, highlighting their relevance as a central element in this signaling cascade. The different G protein families couple to different GPCRs and also initiate distinct downstream signaling pathways. G_s_ proteins stimulate adenylate cyclase, while G_i_ proteins inhibit this enzyme. This influence on the intracellular cyclic adenosine monophosphate (cAMP) levels regulates further downstream effects. G_q/11_ proteins signal *via* phospholipase C (PLC), which cleaves phosphatidylinositol 4,5-bisphosphate (PIP_2_), leading to the release of the second messengers inositol trisphosphate (IP_3_) and diacyl glycerol (DAG), while G_12/13_ proteins activate Rho guanine nucleotide exchange factors (GEFs) leading to remodeling of the cytoskeleton.^5,6^

Further investigation of GPCR signaling pathways is also supported by the ability to selectively regulate sections of those pathways.^7,8^ Selective inhibition of specific G protein families, among other approaches, helps to elucidate the often complex and interconnected cellular signaling events linked to GPCR activation.^9^ One such selective inhibitor is the cyclic depsipeptide FR900359 (FR), which was isolated from the plant *Ardisia crenata*^10^ and has been found to be produced by the plant's endosymbiont *Candidatus* Burkholderia crenata.^11,12^ FR acts as a guanine nucleotide dissociation inhibitor (GDI) by blocking the exchange of GDP for GTP upon GPCR-mediated G protein activation.^13^ It binds selectively to Gα_q_ proteins at the hinge region between the alpha-helical and the GTP-binding domain. FR blocks the conformational changes that accompany nucleotide exchange, thereby keeping the protein in the GDP-bound inactive state and inhibiting further downstream signaling (Fig. 1, bottom). FR is highly selective for the G_q/11_ family and shows a longer residence time than the structurally similar G_q_ inhibitor YM-254890 (YM).^14–17^ Additionally, FR biosynthesis has been established in the cultivable bacterium *Chromobacterium vaccinii* MWU205,^18^ making this molecule a suitable tool to study G_q_-related signaling pathways, including the effects of PLC signaling or second messenger effects of IP_3_ and DAG. Previous studies have shown that FR-mediated G_q_ inhibition could be therapeutically relevant, for example in asthma treatment^15,19^ or uveal melanoma,^20,21^ as the inhibition also affects the GTPase-deficient G_q_ mutant Q209L, which is a hallmark of this type of cancer.^22^

An X-ray crystal structure of YM bound to a chimeric G_i/q_ protein with the N-terminal helix of Gα_i1_ provided structural information about the binding mode of the cyclic depsipeptide inhibitor between linker 1 and switch I.^23^ Further analysis by nuclear magnetic resonance (NMR) spectroscopy of FR and YM in solution highlighted the solvent-dependent presence of *cis*/*trans* isomers with different proposed binding affinities.^24,25^ The development of FR- and YM-radioligands^14^ enabled kinetic studies, pointing to a binding mode of the inhibitor to Gα_q_*via* conformational selection, and highlighting the role of hydrophobic interactions with the isopropyl anchor 2 in FR, leading to a longer residence time compared to YM.^16,17^ Supported by molecular dynamics (MD) simulations^13,25^ the current model for FR-mediated locking of the protein in the GDP-bound form has been proposed, involving rigidification of various parts of the Gα domain upon inhibitor binding, which is yet to be experimentally verified. It has also been shown that FR-insensitive G proteins of different families have similar binding sites as G_q_, which can be turned into FR-binding sites by certain mutations that stabilize the hydrophobic interaction network of the inhibitor.^21,26,27^ This greatly enhances the ability of FR to act as a tool to study G protein signaling more broadly.

In this study we investigate ^13^C^15^N isotope-labeled FR in solution by liquid-state NMR (L-NMR) spectroscopy and provide an extensive resonance assignment in aqueous solution. We compare this solubilized state with the G_q_ heterotrimer-bound state and observe the dynamics of the bound inhibitor in the native-like environment of the lipid membrane, which plays a vital part in G protein function^28^ by solid-state NMR (ssNMR) spectroscopy. In addition, we investigate membrane penetration of FR to understand the access route to the cytoplasmic site of the plasma membrane. Finally, we utilize dynamic nuclear polarization (DNP)-enhanced ssNMR and a unique pair labeling approach to study site-specific effects of inhibitor and nucleotide binding in a FR-sensitive mutant of Gα_i_.

## Experimental

### Preparation of isotope-labeled FR

For large-scale production of isotope-labeled FR, *Chromobacterium vaccinii* MWU205 (German Collection of Microorganisms and Cell Cultures GmbH) was grown in M9 minimal medium (33.7 mM Na_2_HPO_4_, 22 mM KH_2_PO_4_, 8.55 mM NaCl, 9.35 mM ^15^NH_4_Cl, 21.49 mM U-^13^C-glucose, 1 mM MgSO_4_, 0.3 mM CaCl_2_, 134 μM EDTA, 31 μM FeCl_3_, 6.2 μM ZnCl_2_, 0.76 μM CuCl_2_, 0.42 μM CoCl_2_, 1.62 μM H_3_BO_3_, and 0.081 μM MnCl_2_) supplemented with 50 μg mL^−1^ carbenicillin. For the optimized M9 culture medium for FR production, 5 mM of U-^13^C-propionic acid (pH 7) were additionally added. Cultivation was performed at 25 °C for 48 h. Extraction was performed with *n*-butanol at a 1 : 1 ratio over night at 180 rpm. After centrifugation at 4000 rpm for 10–15 min, the upper phase of the extract was collected and the solvent was removed by evaporation. The extract was weighed. If not mentioned otherwise, the samples were diluted with LC/MS grade methanol to 1 mg mL^−1^ for measurement with LC-MS/MS.

LC-MS/MS data were recorded on a micrOTOF-QII mass spectrometer (Bruker) with ESI-source coupled with a HPLC Dionex Ultimate 3000 (Thermo Scientific) using an EC10/2 Nucleoshell C_18_ 2.7 μm column (Macherey-Nagel). The column temperature was 25 °C. MS data were acquired over a range from 100 to 3000 *m*/*z* in positive mode. Auto MS/MS fragmentation was achieved with rising collision energy (35–50 keV over a gradient from *m*/*z* 500–2000) with a frequency of 4 Hz for all ions over a threshold of 100. High-performance liquid chromatography (HPLC) begins with 90% H_2_O containing 0.1% AcOH. The gradient starts after 1 min to 100% acetonitrile (0.1% AcOH) in 20 min. A 5 μL amount of a 1 mg mL^−1^ sample solution (MeOH) was injected to a flow of 0.3 mL min^−1^. Data analysis was performed using Bruker Compass DataAnalysis Version 4.2 (Build: 383.1).

For isolation of uniformly ^13^C^15^N-labeled FR, the crude butanolic extract was fractionated on a Grace Reveleris X2 flash chromatography system with integrated evaporative light scattering (ELSD) and UV-Vis detection *via* a Reveleris C_18_ flash column (220 g, 40 μm). A stepwise gradient solvent system of increasing polarity and a flow rate of 65 mL min^−1^ was used starting with 50/50 H_2_O/MeOH for 13 min, then changing to 30/70 H_2_O/MeOH within 1 min and held again for 13 min. The gradient was then changed within 1 min to 25/75 H_2_O/MeOH and held for 22 min, then within 1 min to 20/80 H_2_O/MeOH, held for 13 min, then within 1 min to 15/85 H_2_O/MeOH and held for 15 min. Finally, the gradient was changed within 1 min to 100% MeOH and held for an additional 10 min. According to the measured ELSD and UV signals, a FR containing fraction was collected at 70 min. Final purification was done by HPLC with a semi-preparative Macherey-Nagel Nucleodur C_18_ column (250 × 8 mm, 5 μm) using an isocratic elution with 20/80 H_2_O/MeOH (flow 2.0 mL min^−1^). Uniformly ^13^C^15^N-labeled FR was isolated as a white powder (t_R_: 20 min, 40 mg).

For the small-scale cultures to examine the impact of propionic acid on the FR/FR-2 production ratio, *C. vaccinii* was grown in unlabeled M9 minimal medium (composition as mentioned above) supplemented with 50 μg mL^−1^ carbenicillin to avoid contamination. Three cultures with 50 mL M9 medium were inoculated with *C. vaccinii* and supplemented with 5 mM propionic acid (pH 7), three cultures were inoculated with *C. vaccinii* but not supplemented, and three cultures were not inoculated. Cultivation was performed at 25 °C for 48 h. Extraction was performed 1 : 1 with *n*-butanol over night at 180 rpm. Afterwards, the upper phase was collected using centrifugation at 4000 rpm for 10–15 min followed by evaporation. The extract was weighed. If not mentioned otherwise, the concentration of samples diluted with LC/MS grade methanol was 1 mg mL^−1^ for measurement with LC/MS. The average AUC for *m*/*z* 988.5 and *m*/*z* 1002.5 of the controls were deducted from the AUC of each inoculated sample.

High-performance liquid chromatography mass spectrometry (HPLC/MS) data was recorded on a Waters 2695 separation module, which was coupled to a Waters 996 photodiode array detector, and a Waters QDa detector with electrospray ionization source. For separation a gradient elution with mobile phases A (acetonitrile/water 5/95 with 5 mM ammonium acetate and 40 μL acetic acid per Liter) and B (acetonitrile/water 95/5 with 5 mM ammonium acetate and 40 μL acetic acid per liter) on a Waters X Bridge Shield RP_18_ column (100 × 2.1 mm; 3.5 μm) at 25 °C were used (flow of 0.3 mL min^−1^, 80/20 A/B to 0/100 A/B within 20 min and hold for 10 min). MS data was collected in positive and negative mode in the range between *m*/*z* 140–1250 and additionally in the positive single ion mode for the mass trace of FR (*m*/*z* 1002.5; M + H^+^). HPLC was carried out either using a Waters HPLC system, controlled by Waters Millennium software, consisting of a 600E pump, a 996 PDA detector, and a 717 plus autosampler or on a Waters Breeze HPLC system equipped with a 1525μ dual pump, a 2998 photodiode array detector, and a Rheodyne 7725i injection system.

### Insect cell expression of G_q_

For the expression of the G_q_ heterotrimer, three baculoviruses generated by the BestBac method (Expression Systems) were coinfected encoding the wild-type human Gα_q_ subunit, the wild-type human Gβ_1_γ_2_ subunits, and the rat guanine nucleotide exchange factor Ric-8A. To facilitate purification of the G_q_ heterotrimer, a His_6_-tag with an HRV-3C protease cleavage site was attached at the amino terminus of the Gβ_1_ subunit. *Trichoplusia ni* cells (Expression Systems) were infected with the baculoviruses at a cell density of 3 mill per mL followed by incubation of 48 hours at 27 °C. After harvest by centrifugation, cells were lysed in a hypotonic buffer composed of 10 mM Tris, pH 7.5, 100 μM MgCl_2_, 5 mM β-ME, 10 μM GDP and protease inhibitors (20 μg mL^−1^ leupeptin, 160 μg mL^−1^ benzamidine). The membrane fraction was collected by centrifugation and solubilized with 20 mM HEPES, pH 7.5, 100 mM NaCl, 1% Na-cholate, 0.05% DDM, 5 mM MgCl_2_, 5 mM β-ME, 5 mM imidazole, 10 μM GDP and protease inhibitors. After homogenization with a Dounce homogenizer, the solubilization reaction was incubated for 45 min at 4 °C. After centrifugation, the soluble fraction was loaded onto Ni^2+^-chelating Sepharose (Cytiva, Munich, Germany) followed by a gradual detergent exchange into 0.1% DDM. The protein was eluted in buffer supplemented with 200 mM imidazole and dialyzed overnight against 20 mM HEPES, pH 7.5, 100 mM NaCl, 0.1% DDM, 1 mM MgCl_2_, 5 mM β-ME and 10 μM GDP together with HRV-3C protease to cleave off the amino-terminal His_6_-tag. Cleaved His_6_-tag, uncleaved fractions and HRV-3C protease were removed by Ni^2+^-chelating Sepharose. The cleaved G protein was dephosphorylated by lambda protein phosphatase (NEB, Frankfurt, Germany), calf intestinal phosphatase (NEB, Frankfurt, Germany), and antarctic phosphatase (NEB, Frankfurt, Germany) in the presence of 1 mM MnCl_2_. Lipidated G_q_ heterotrimer was isolated using a MonoQ 5/50 GL column (Cytiva, Munich, Germany). The protein was bound to the column in buffer A (20 mM HEPES, pH 7.5, 50 mM NaCl, 1 mM MgCl_2_, 0.1% DDM, 100 μM TCEP, 10 μM GDP) and washed in buffer A. The G_q_ heterotrimer was eluted with a linear gradient of 0 to 50% buffer B (buffer A + 1 M NaCl). The main peak containing isoprenylated G_q_ heterotrimer was collected and dialyzed against 20 mM HEPES, pH 7.5, 100 mM NaCl, 0.1% DDM, 100 μM TCEP and 10 μM GDP. The protein was concentrated to 26 mg mL^−1^, 20% glycerol was added, and the protein was flash frozen in liquid nitrogen and stored at −80 °C until use.

### G_q_ protein reconstitution

Liposomes for the reconstitution of the G_q_ heterotrimer were prepared from POPC, POPE, and cholesterol (Avanti Polar Lipids). The lipid powder was weighed in a 5 : 4 : 1 molar ratio and dissolved in chloroform : methanol 1 : 1 (v/v) to ensure proper mixing. The dissolved lipids were dried overnight on a rotary evaporator at 80 mbar, followed by two hours at 10 mbar to remove the solvents and form a lipid film. This film was resuspended in lipid buffer (20 mM HEPES pH 7.5, 100 mM NaCl) for 1 hour at 42 °C. The lipid suspension was subjected to 5 freeze–thaw cycles and subsequent 11 extrusion cycles through a 0.4 μm membrane to form evenly sized liposomes. The liposomes were used right away for reconstitution or stored at −20 °C. Freshly thawed liposomes were re-extruded before usage.

For reconstitution, the G_q_ protein was diluted with G_q_ buffer (20 mM HEPES pH 7.5, 100 mM NaCl, 50 μM GDP, 100 μM TCEP, 1 mM MgCl_2_, 0.1% DDM) to a concentration of 1 mg mL^−1^. The protein solution was mixed with the extruded liposome solution at a molar lipid-to-protein ratio (LPR) of 250 : 1 and stirred for 30 minutes at room temperature. After reconstitution, the detergent was removed by rotating incubation of the sample with bio-beads (Bio-Rad) over night at 4 °C. Once the proteoliposome solution turned turbid again, the bio-beads were removed and the sample was pelleted by ultracentrifugation (30.000×*g*, 30 minutes). The sample was resuspended in 1 mL lipid buffer and for paramagnetic NMR-doping, 10 μL Gd^3+^-DOTA [100 mM] was added. After rotating incubation for 30 minutes at 4 °C, the sample was ultracentrifuged again (222.600×*g*, 30 minutes) to create a dense NMR pellet that was centrifuged into a 3.2 mm or 4 mm NMR-rotor for ssNMR experiments.

### *E. coli* expression of Gα_i/q_

The expression plasmid for Gα_i/q_ was generated by subcloning the sequence of the mutated *GNAI1* gene from an insect cell vector into the pET16b vector for *E. coli* expression. The gene carried the mutations V50I, K54R, Y69F, V72L, K180P, V185I, T187Y, and H188P necessary for sensitivity of the protein towards FR. Subcloning was performed by amplifying the target gene *via* PCR and digesting the insert as well as the target plasmid with the restriction enzymes NdeI and XhoI (Thermo Fisher Scientific). Subsequent ligation and transformation into DH5α cells (New England Biolabs GmbH) allowed the confirmation of the subcloned sequence by plasmid isolation and sequencing. In the *E. coli* expression system, Gα_i/q_ was N-terminally tagged by a 10x His-Tag connected to the protein by a 10 aa linker and put under the expression control of the *lac* operon. Additionally, the *bla* gene, leading to ampicillin resistance, on the pET16b vector was used for successful transformant selection.

Protein expression was conducted by transforming the expression plasmid into chemically competent *E. coli* BL21 cells (Thermo Fisher Scientific) *via* heat shock. After plating on ampicillin-containing agar plates and incubation over night at 37 °C, a single colony was picked and added to 100 mL LB-medium with 0.1 mg mL^−1^ ampicillin as preculture. The preculture was grown overnight at 37 °C at an agitation of 180 rpm. The main culture consisted of 1 L TB-medium with 0.1 mg mL^−1^ ampicillin and was inoculated with 10 mL of preculture. During incubation at 37 °C and agitation at 220 rpm the optical density at 600 nm (OD_600_) was measured to monitor bacterial growth. At an OD_600_ of 0.6 the cultures were harvested by centrifugation (6400×*g*, 10 minutes) and remains of the TB-medium were washed away by resuspending the cells in minimal M9-medium and repeated centrifugation. The washed cell pellet was resuspended in and added to 600 mL expression medium of defined M9 medium with different supplements, depending on the chosen NMR-labeling scheme for the sample. For unlabeled protein expression, defined M9-medium was used, supplemented with amino acids, glucose and NH_4_Cl. For specifically labeled samples, the used amino acids were exchanged for their labeled counterparts. ^13^C-valine (230 mg L^−1^) and ^15^N-proline (100 mg L^−1^) were used for the VP-labeled sample, ^13^C^15^N-arginine (400 mg L^−1^) and ^15^N-methionine (250 mg L^−1^) were used for the RM-labeled sample and ^13^C-valine (230 mg L^−1^) and ^15^N-phenylalanine (130 mg L^−1^) were used for the VF-labeled sample. For the latter, 4-Hydroxyphenylpyruvic acid (800 mg L^−1^), a precursor for tyrosine, was also added to prevent metabolic scrambling.^29^ After adaptation to the expression medium for 1 hour at 37 °C at an agitation of 220 rpm, the expression was induced by the addition of 1 mM isopropyl β-d-1-thiogalactopyranoside (IPTG) to the medium. After induction, the culture was kept at 20 °C for 17 hours at an agitation of 250 rpm. The cells were harvested by centrifugation (6400×*g*, 10 minutes) and stored at −80 °C or used for protein purification.

For lysis, the cell pellet was dissolved in buffer A (25 mM Tris pH 7.5, 0.5 M NaCl, 5 mM TCEP, 50 mM imidazole, 10% (v/v) glycerol) and passed four times through a cell disruptor at 1.8 bar. The lysed cells were ultracentrifuged (250.000×*g*, 1 hour) to remove debris and cellular membranes and the supernatant was incubated two hours at 4 °C with buffer A pre-equilibrated TALON cobalt-containing metal affinity resin (Takara Bio) for batch binding and immobilized metal affinity chromatography. After binding, the resin was added to a gravity flow column and washed with 20 column volumes (CV) buffer A to remove unspecifically bound protein from the column. For elution the column was incubated 2 times with 3 CV buffer B (25 mM Tris pH 7.5, 0.5 M NaCl, 5 mM TCEP, 500 mM imidazole, 10% (v/v) glycerol). The eluate was pooled and concentrated with a 10.000 MWCO concentrator (Merck) for buffer exchange to buffer C (25 mM HEPES pH 7.5, 0.15 M NaCl, 2 mM DTT).

### Biochemical characterization

Protein concentration was determined *via* absorption measurements at 280 nm conducted at a nanodrop spectrophotometer with an expected protein mass of 44.01 kDa and an extinction coefficient of 35.870 M^−1^ cm^−1^ for Gα_i/q_. Further analysis and purification by size exclusion chromatography was performed on a Bio-Rad NGC chromatography system with a Superdex 200 Increase 10/300 GL column (GE Healthcare). Protein-containing fractions were identified by their absorption at 280 nm and collected for further experiments. For SDS-PAGE analysis the protein samples were diluted with 4x SDS-PAGE sample buffer (62.5 mM Tris pH 6.8, 0.1 g L^−1^ LDS, 100 g L^−1^ glycerol, 255 mM β-mercaptoethanol, 50 mg L^−1^ bromophenol blue) and 20 μL were loaded into a SurePAGE 4–12% gel (as well as 5 μL of protein marker (PageRuler Prestained Protein Ladder, Thermo Scientific). The gel was run in SDS running buffer (50 mM MES, 50 mM Tris, 1 g L^−1^ SDS, 1 mM EDTA) for 50 minutes at 150 V for protein band separation. For the detection of the Gγ subunit a 4–20% Mini-PROTEAN gel (Bio-Rad) was run in Rotiphorese buffer (Roth). The gel was stained overnight in staining solution (20% ethanol, 10% glacial acetic acid, 2.5 g L^−1^ Coomassie R-250) and destained for two hours in destaining solution (20% ethanol, 10% glacial acetic acid).

### Radioligand binding studies

The purified chimeric Gα_i/q_ protein was preincubated with TALON immobilized metal affinity chromatography (IMAC) resin beads (Takara Bio) for 1–2 h at 4 °C with gentle shaking (0.025 μL beads per 0.1 mL of protein solution in 50 mM Tris–HCl, pH 7.4; final protein amount per sample: 5 μg). Each sample contained 70 μL of assay buffer (50 mM Tris–HCl, pH 7.4), 5 μL of dimethyl sulfoxide (DMSO) or the test compound (dissolved in DMSO), 50 μL of [^3^H]PSB-15900 (FR-derived radioligand; 28 Ci/mmol; Pharmaron, Cardiff, UK)^14^ in assay buffer (final concentration: 5 nM) and 125 μL of protein solution. Total binding of [^3^H]PSB-15900 was determined using 5 μL of DMSO, and non-specific binding was determined using 5 μL of unlabeled FR900359 (final concentration: 5 μM). For competition binding experiments, dilutions from 30 μM to 0.003 μM of unlabeled FR900359, dissolved in DMSO, were added. Incubation was performed for 2 h at 4 °C with gentle shaking. Incubation was terminated by rapid filtration through Whatman GF/C glass fiber filters (GE Healthcare Life Sciences Whatman™, USA) and washing with 4 × 3 mL of ice-cold buffer containing 50 mM Tris–HCl (pH 7.4), 0.1% of bovine serum albumin (BSA), and 0.1% of Tween 20. After punching out the filters, 2.5 mL of scintillation cocktail (ProSafe FC+, Meridian Biotechnologies Ltd, Surrey, UK) were added and the samples were directly measured using a liquid scintillation counter. Data analysis was carried out with GraphPad Prism version 8.0. For calculating *K*_D_- and *B*_max_-values, the one-site homologous equation was used. Data represent means ± standard deviation of three independent experiments, performed in duplicates.

### Liquid-state NMR experiments

Assignment experiments were recorded on ^13^C^15^N-labeled FR [11.65 mM] dissolved in G_q_ buffer with the addition of 5% D_2_O and 0.15 mM 2,2-dimethyl-2-silapentan-5-sulfonat (DSS) for referencing. The sample was recorded at an 800 MHz Avance III HD spectrometer with a 5 mm 1H{^13^C/^15^N} TCI CryoProbe (Bruker) at a temperature of 298 K. 2D ^15^N-^1^H-BEST-TROSY, 2D ^15^N-^1^H-TROSY-H(N)CACB, 2D ^15^N-^1^H-TROSY-H(N)COC, 2D ^15^N-^1^H-TROSY-H(NCA)CO, 2D ^13^C-^1^H-HSQC, 2D ^13^C-detected CON, 2D ^13^C-detected (HCA)CON, 3D HCN and 3D (H)CCH-TOCSY were recorded for FR resonance assignment. Solvent effects were studied on a sample of the ^13^C^15^N-labeled FR dissolved in either G_q_ buffer with the addition of 5% D_2_O and 0.15 mM DSS for referencing or CDCl_3_ referenced to the solvent. The sample in aqueous G_q_ buffer was recorded at an 800 MHz spectrometer, the CDCl_3_-dissolved sample at a 1.2 GHz Avance Neo spectrometer with a 3 mm ^1^H{^13^C/^15^N} TCI CryoProbe (Bruker). For both samples 2D ^15^N-^1^H-BEST-TROSY spectra were recorded. ^1^H-^1^H-NOESY spectra of ^13^C^15^N-FR in G_q_ buffer without detergent to avoid detection of FR-detergent NOE signals were recorded at 1.2 GHz and 298 K with different mixing times (50/100/200/400/800 ms) with referencing to DSS. A sample of the same composition was used to study dynamics of FR in solution at 298 K. At a 600 MHz spectrometer ^15^N T_1_ and T_2_ relaxation times were determined by gradient-selected, sensitivity enhanced HSQC-based experiments and picosecond dynamics were investigated by recording a TROSY version of a {^1^H}^15^N-hetNOE experiment. The contribution of chemical exchange was determined by recording ^15^N-CPMG relaxation dispersion experiments at both 600 and 950 MHz ^1^H frequencies. The data was analyzed with Dynamics Center 2.8.5 (Bruker) and the Carver Richards Model was utilized to fit the data.^30^

### Solid-state NMR experiments

In order to study binding of FR to liposome membranes and the G_q_ protein, two samples were prepared. The first sample consisted of extruded POPC : POPE : Cholesterol (5 : 4 : 1) liposomes, while the second sample contained 836 μg G_q_ protein reconstituted into the same liposome composition at a molar LPR of 250 : 1. The samples were pelleted into a 3.2 mm magic-angle spinning (MAS) rotor and in order to study FR binding, ^13^C^15^N-labeled FR from a 30 mM stock solution in d_6_-DMSO was added directly into the rotor at a 2 : 1 molar surplus over the reconstituted protein. The experiments were conducted at a nominal temperature of 290 K for liposomes in the liquid-crystalline phase and at a nominal temperature of 260 K for the gel phase. Since the sample temperature inside the rotor under MAS conditions can be higher, a more precise sample temperature during the experiment was calculated based on the chemical shift of the H_2_O signal.^31^ Based on this calculation the nominal temperatures of 260 K and 290 K corresponded to 275 K and 305 K, respectively. A 600 MHz Avance Neo spectrometer (Bruker) with a 3.2 mm MAS HCN efree probe (Bruker) was used for the experiment and a MAS rate of 14 kHz was chosen. ^13^C-CP with 83.33 kHz proton decoupling and ^13^C-INEPT experiments were recorded.

To assess the membrane penetration depth, ^13^C^15^N-labeled FR was also added to POPC : POPE : Cholesterol liposomes and ^1^H-^1^H-NOESY spectra with different mixing times (50/100/200/400/800 ms) were recorded before and after the addition of FR. A MAS rate of 14 kHz at a 600 MHz spectrometer and nominal temperatures of 290 K for the liquid-crystalline phase and 260 K for the gel phase were chosen. Spin-Lattice relaxation times T_1_ in the NOESY analysis were determined by an exponential decay fit of the diagonal aromatic signal for the different mixing times.

Two samples of the same makeup as the samples to study FR binding to membranes with or without G_q_ protein were used to study GTP hydrolysis and hydrolysis inhibition by FR at a nominal temperature of 310 K, corresponding to a calculated sample temperature of 326 K, in the liquid-crystalline phase. Those experiments were conducted with a 4 mm rotor at a 600 MHz spectrometer with a 4 mm MAS HX probe (Bruker) and a MAS rate of 8 kHz was chosen. GTP-hydrolysis was observed by ^31^P-detection with 100 kHz proton decoupling over a 48 hour spanning series of experiments after the addition of 5 μL of a 17 mM solution of GTP : MgCl_2_ (1 : 1 molar ratio). Quantification of β-GTP peaks was done by fitting of the measured β-GTP signal in Origin 2017 with the non-linear Lorentz peak function. The fit error for the peak area was given as error in the respective figure.

Assignment experiments were recorded on a sample of 5 mg reconstituted G_q_ protein incubated with ^13^C^15^N-labeled FR from a 30 mM stock solution in d_6_-DMSO (2 : 1 molar surplus) and subsequently washed two times with lipid buffer. The experiments were conducted at a nominal temperature of 260 K for liposomes in the gel phase. For the ^13^C-assignment, a set of 2D ^13^C^13^C-PDSD experiments was recorded with 20/50/200 ms mixing time and for the ^15^N-assigment, 2D HNCO (0.8/3 ms ^15^N-CP contact time) and 2D HNCA (0.8/3 ms ^15^N-CP contact time) experiments were conducted. All assignment experiments were recorded using a 3.2 mm rotor at a 600 MHz spectrometer with a 3.2 mm MAS HCN efree probe, at a MAS rate of 14 kHz and with 83.33 kHz proton decoupling except for the ^13^C^13^C-PDSD (200 ms mixing time), which was recorded at an 850 MHz Avance III spectrometer (Bruker) with a 3.2 mm MAS HCN efree probe at a MAS rate of 14 kHz and with 83.33 kHz proton decoupling. NMR-spectra were processed using the software Topspin 4.0.9, resonance assignment was supported by CCPN Version 3.^32^ The same sample was also used for 2D ^15^N-SLF (0.8 ms ^15^N-CP contact time) experiments at identical measurement conditions to determine NH dipolar couplings of G_q_-bound ^13^C^15^N-labeled FR. Due to the chosen MAS frequency of 14 kHz the R18^5^_2_ recoupling sequence was applied at 63 kHz. The total echo time was set to 5 ms and 6.6 ms acquisition time was chosen for the indirect dimension. The dipolar coupling was obtained from the peak splitting using the theoretical scaling factor for the applied R18_2_^5^ recoupling sequence of 0.3038.^33^ The dipolar order parameter was calculated from the ratio between the measured dipolar coupling and the theoretical NH dipolar coupling value of 11 648 Hz, corresponding to an effective bond length of 1.015 Å.^34,35^

For all samples the phase of the lipid bilayer was determined from the observed ^1^H line width of the lipid signals. The ^1^H and ^13^C referencing in all MAS-NMR experiments was done indirectly to DSS *via*^13^C-CP on 1–^13^C-alanine at 179.85 ppm. ^31^P referencing was done indirectly to 10% trimethyl phosphate using the ^31^P signal of triethylphosphine sulfide (TEPS) at 55.58 ppm.

### DNP experiments

The protein response to inhibitor binding was studied by HNCO(CX) experiments with 20 ms mixing time recorded on DNP samples of frozen Gα_i/q_ protein solution. The polarizing agent AsymPolPOK^36^ was dried from a chloroform : methanol (1 : 1) stock solution to form a film that was resuspended with 1 mg of protein solution that was concentrated to a volume of 15 μL, subsequently 15 μL of d_8_-glycerol was added. The final concentration of radical in the mixture with the protein sample in the 3.2 mm NMR rotor was 10 mM. The samples were measured at a DNP setup consisting of a 400 MHz Avance Neo spectrometer (Bruker) with a 3.2 mm Cryo MAS HCN probe (Bruker) and a 263 GHz Gyrotron as microwave source. All experiments were done at a nominal temperature of 105 K. A MAS rate of 8 kHz and proton decoupling of 100 kHz were chosen. To investigate the effects of inhibitor binding, the protein solution was incubated with unlabeled FR or YM from a 10 mM DMSO stock solution at an inhibitor to protein molar ratio of 1.5 : 1 at 4 °C overnight. To study the effects of nucleotide binding, 5 μL of GDP was added to the samples from a 17 mM solution of GDP : MgCl_2_ (3 : 1 molar ratio) directly into the rotor. The GTP-bound state was generated by subsequent addition of 2 μL of GTP [0.1 M] and 2 μL AlF_4_ salt solution (150 mM MgCl_2_, 75 mM NaF, 15 mM AlCl_3_). DNP enhancement was measured by comparison of ^13^C-CP spectra with and without microwave irradiation. Referencing was done indirectly using the right peak of d_8_-glycerol at 64.78 ppm.

## Results and discussion

### Preparation of isotope-labeled FR

**Fig. 2:**
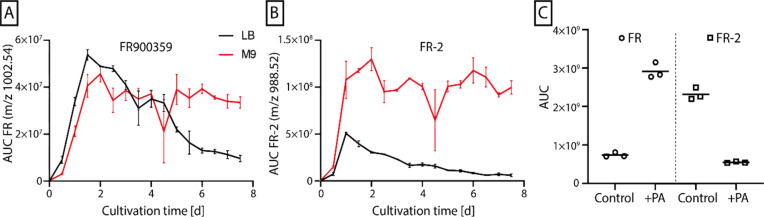
Production of FR900359 measured *via* LC/MS as AUC for the *m*/*z* 1002.54 (A) and FR-2 measured as AUC for the *m*/*z* 988.52 (B) by *Chromobacterium vaccinii* MWU205 cultivated in LB medium (black) and M9 medium (red) over 7.5 d. Error bars show the standard deviation of two repeats. (C) Comparison of FR/FR-2 production cultures in M9 medium without (control) and with addition of 5 mM propionic acid (+PA). Experiments were performed three times.

To conduct a broader range of more complex NMR experiments on FR, isotope labeling of this compound was required. FR production by *Chromobacterium vaccinii* MWU205 in a chemically defined minimal medium (M9) was established.^37^ Subsequent exchange of the nitrogen source NH_4_Cl for ^15^NH_4_Cl and the carbon source glucose for the uniformly ^13^C-labeled glucose enabled the production of uniformly ^13^C^15^N-labeled FR. A first cultivation experiment followed by *n*-butanol extraction, fractionation *via* flash chromatography, and investigation *via* liquid chromatography-mass spectrometry (LC-MS/MS) (Fig. S1†) verified the successful isotope incorporation and generation of uniformly ^13^C^15^N-labeled FR. However, a large-scale 10 L cultivation experiment only yielded 3 mg ^13^C^15^N-FR, at the same time a considerable amount of 55 mg ^13^C^15^N-FR-2 was purified. This FR-derivative features *N*-acetyl-β-hydroxyleucine instead of *N*-propionyl-β-hydroxyleucine as side chain (Fig. S1 B†). FR/FR-2 production titers showed similar responses when comparing *C. vaccinii* in complex lysogeny broth (LB) medium and chemically defined minimal M9 medium. In both media, the production peaked after 36 h of cultivation but afterwards the amount of FR and FR-2 decreased in LB medium, while its production in M9 medium remained stable over the course of 7.5 days. While the production titer curve shape was similar for both compounds, the produced amount, indicated by the area under the curve (AUC), was higher for FR-2 in M9 medium compared to FR (Fig. 2A and B).

Since the only difference between the compounds was the incorporation of acetic acid in FR-2 instead of propionic acid (PA) in FR, the observed deviating production levels could be due to a different abundance of PA in the applied media. This was explored in a small-scale experiment with *C. vaccinii* grown in M9 medium with added PA. Analysis of *n*-butanol extracts by LC/MS depicted an increase of FR production due to the addition of PA, while FR-2 production levels did decrease (Fig. 2C). This suggested a lower production of PA by *C. vaccinii* in M9 medium, causing the difference in FR/FR-2 production levels. After optimization by addition of ^13^C-labeled PA to 4.5 L isotope-labeled culture medium, the production of uniformly ^13^C^15^N-labeled FR was enhanced and 40 mg of the labeled compound were purified.

### FR resonance assignment in aqueous solution

Uniformly ^13^C^15^N-labeled FR was analyzed by L-NMR to confirm successful isotope labeling and assign ^1^H, ^13^C and ^15^N resonances. This analysis was conducted in aqueous buffer, which corresponds to the G_q_ protein buffer to allow for comparison between the chemical shifts in solvent and G_q_-bound FR. The obtained ^1^H chemical shift were overall comparable with the chemical shifts in H_2_O/D_2_O determined in a previous study^24^ showing only a small uniform perturbation due to the change in buffer. Differences were larger for ^13^C chemical shifts as they had been determined in organic solvent only. Due to the isotope labeling employed here, also ^15^N chemical shifts of FR could be determined. Also, ^1^H-^15^N correlation spectra of FR in various solvents (aqueous buffer, CDCl_3_) showed similar sensitivity of the chemical shifts towards the chosen solvent, leading to strong solvent dependent signal shifts (Fig. S2†), highlighting the necessity to choose the appropriate solvent to allow for chemical shift perturbation (CSP) analysis due to protein binding. The four protonated nitrogen atoms were visible in [^15^N, ^1^H]-BEST-TROSY spectra (Fig. S3†) together with weaker signals from isotope labeled co-purified impurities. A combination of HCN- and CON-experiments allowed for the detection of three methylated nitrogen residues. In total, all ^1^H and ^15^N and 47 of 49 ^13^C resonances were assigned in aqueous buffer (Table S1†).

### Expression and reconstitution of the G_q_ heterotrimer

The wild-type human G protein G_q_ was expressed in *Trichoplusia ni* (*Tni*) insect cells using a baculovirus expression system to purify lipid-modified, heterotrimeric G protein capable of associating with the plasma membrane. G_q_ possesses three lipid moieties: two *S*-palmitoylations at the N-terminal cysteine residues C9 and C10 of the Gα_q_ subunit, and geranylgeranylation at the C-terminal cysteine C68 of the Gγ_2_ subunit. These post-translational modifications have been shown to be important for membrane targeting of the G protein heterotrimer.^38,39^ To increase the yield of functional G_q_ heterotrimer, it was co-expressed with the rat glutathione *S*-transferase (GST)-tagged GEF resistance to inhibitors of cholinesterase 8A (Ric-8A), known to fold nascent Gα subunits (Gα_i/o_, Gα_12/13_ and Gα_q/11_) prior to G protein heterotrimer formation.^40,41^ The G_q_ heterotrimer was subsequently solubilized from the membrane of the *Tni* cells using detergents sodium cholate and *n*-dodecyl-β-d-maltoside (DDM) and purified in DDM to homogeneity using Ni^2+^-affinity and ion exchange chromatography. The purified protein was analyzed by sodium dodecyl sulfate-polyacrylamide gel electrophoresis (SDS-PAGE) and gel filtration (Fig. S4 A and B†).

1-palmitoyl-2-oleoyl-*glycero*-3-phosphocholine (POPC): 1-palmitoyl-2-oleoyl-*sn-glycero*-3-phosphoethanolamine (POPE) : Cholesterol (5 : 4 : 1 mol%) liposomes were chosen as suitable membrane environment for the heterotrimeric G protein to conduct ssNMR measurements, reflecting the high amount of PE in the inner leaflet of the plasma membrane, where G_q_ is located *in vivo*.^42^ Cholesterol was added as it is an important constituent of mammalian membranes and effects lipid dynamics and phase transition behavior. In order to assess successful reconstitution of G_q_ heterotrimer into these liposomes, several samples were taken during the proteoliposome preparation and analyzed by SDS-PAGE for their protein content (Fig. S4 C†). The two bands at ∼40 kDa correspond to Gα_q_ (42.1 kDa) and Gβ_1_ (37.7 kDa) showing unreconstituted G protein in the supernatant and reconstituted protein in the proteoliposome pellet. The latter gives rise to smeared bands due to the uneven migratory behavior of liposomes compared to lipid-free samples in the gel. No signal was observed in the supernatant of the washing step, thus excluding the presence of loosely liposome-associated protein. The weaker signal for Gγ_2_ (7.9 kDa) could only be detected on higher percentage SDS-PAGE.

### Analysis of G_q_-bound FR in liposomes

**Fig. 3:**
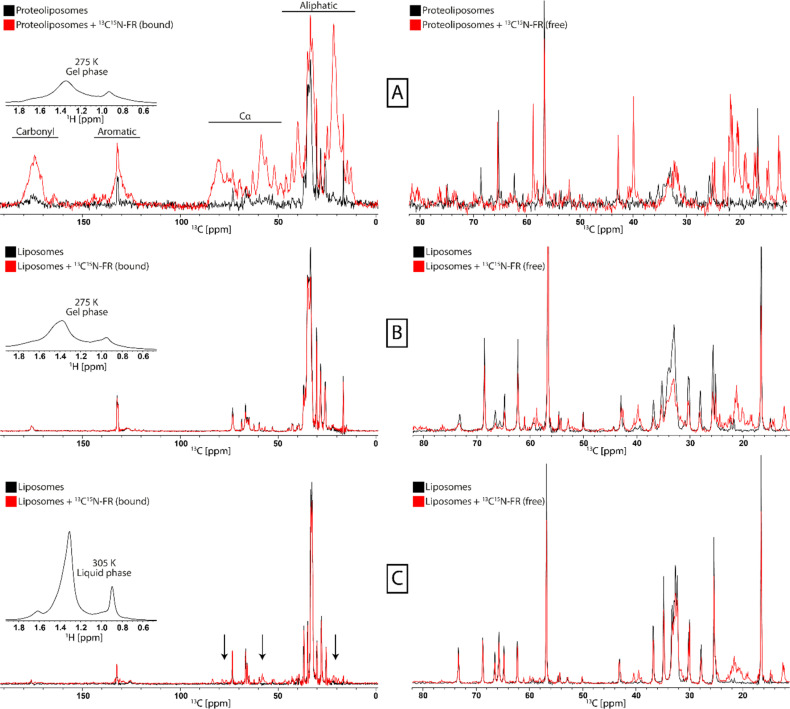
Characterization of interactions between FR and G_q_ proteoliposomes and liposomes by ssNMR. ^13^C-CP spectra for the detection of immobile components (left) and ^13^C-INEPT spectra for the detection of mobile components (rights) were recorded for G_q_ proteoliposomes in the gel phase (A), POPC : POPE : Cholesterol (5 : 4 : 1) liposomes in the gel phase (B), and POPC : POPE : Cholesterol (5 : 4 : 1) liposomes in the liquid-crystalline phase (C). In the presence of ^13^C^15^N-labeled FR (red) additional strong signals of immobilized inhibitor molecules appear only in the G_q_ proteoliposome sample, indicating binding to the protein. In addition, for the liposomes in the liquid-crystalline phase some weak signals also appear, pointing to membrane-embedded FR (arrows). The respective membrane phase is monitored by ^1^H-spectra (insert). The respective CH_2_ resonances are broad in the gel phase and narrow in the liquid crystalline phase.

G_q_ proteoliposomes were incubated with ^13^C^15^N-FR and subsequently MAS ssNMR experiments were recorded. FR was added in surplus and differentiation between bound and free FR was achieved by using cross-polarization (CP) based and insensitive nuclei enhanced by polarization transfer (INEPT) based experiments, respectively.^43,44^ The experiments were conducted in the gel phase without freezing the sample as those conditions allow for effective CP transfer without freeze-immobilization of soluble compounds. Comparison of the ^13^C-CP spectra of the G protein proteoliposomes before and after the addition of ^13^C^15^N-FR showed strong additional signals in the FR-treated sample from immobilized FR (Fig. 3A). In addition, the J-coupling based INEPT spectrum, which selects for signals from mobile components, showed the free FR in both samples that was not immobilized by binding to the protein. In a control experiment with protein-free liposomes, no FR signals were observed in the ^13^C-CP spectra, whereas signals were still present in the INEPT experiment, revealing the absence of unspecific binding of FR to the lipids (Fig. 3B). Interestingly, when the control experiments were repeated at a higher temperature, resulting in the more mobile liquid-crystalline membrane phase of the liposomes, FR gave rise to signals in the ^13^C-CP spectrum, indicating some interaction with the protein-free liposomes (Fig. 3C). A possible explanation for this observation could be that the increased fluidity of the membrane resulted in a more efficient penetration and interaction of FR with the bilayer than in the more densely packed gel phase. As membranes *in vivo* are in the liquid-crystalline phase, this could provide one mode of access for the inhibitor to cross the plasma membrane and reach its G protein target anchored at the inner leaflet of the lipid bilayer.

### FR-membrane interactions

**Fig. 4:**
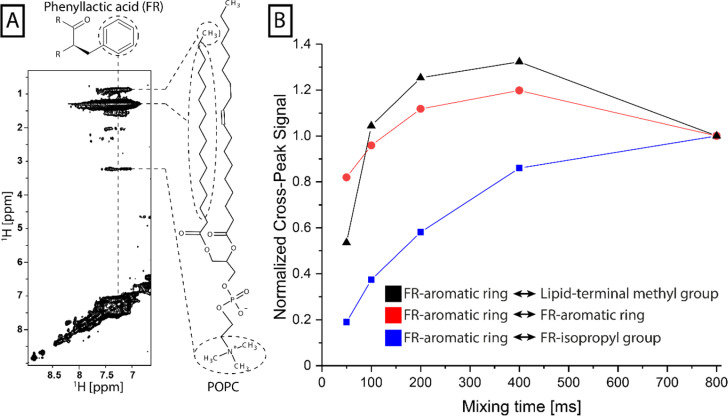
NOE build-up for selected intramolecular FR–FR and intermolecular FR-lipid contacts. (A) The aromatic region of the ssNMR ^1^H-^1^H-NOESY (400 ms mixing time) shows cross-peaks that represent intermolecular contacts of the aromatic ring of the phenyllactic acid moiety of FR to various parts of POPC lipids. (B) The normalized cross-peak intensities of ssNMR and L-NMR NOESY spectra were plotted against the experimental mixing times (50/100/200/400/800 ms). The putative FR-lipid contact (black) shows a fast build-up similar to the short-range NOE curve for contacts inside of the aromatic ring of FR (red) in contrast to the intramolecular FR contact between the aromatic ring and the isopropyl group of the *N*-acetylhydroxyleucine moiety (blue).

To further investigate the interactions between FR and the lipid bilayer in the liquid-crystalline phase, the membrane penetration was assessed by ^1^H-^1^H-NOESY spectra, in which cross-peaks arise from spatially close protons. Here we focused on the cross-peaks arising from the aromatic region (7–8 ppm) of the FR molecule as they do not overlap with any signal from the lipid bilayer (Fig. S5†). Strong cross-peaks were observed in ssNMR ^1^H-^1^H-NOESY experiments between these aromatic protons and the lipid molecules (Fig. 4A), namely the CH_2_ acyl chains (1.3 ppm) and the choline head group (3.24 ppm). Interestingly, also a cross-peak was observed at 0.89 ppm, which could either arise from a contact to the terminal CH_3_ group of the lipid molecules or from intramolecular correlations within the FR molecule from the *N*-acetylhydroxyleucine moiety. This intramolecular correlation was investigated by conducting the same experiments as L-NMR ^1^H-^1^H-NOESY on the FR molecule alone in solution (Fig. S6†). It was determined that this contact was rather long-ranged, corresponding to a slow nuclear Overhauser effect (NOE)-build-up. In contrast, the build-up of the cross-peak at 0.89 ppm in the NOESY spectra recorded on the liposome samples was much faster (Fig. 4B), despite similar T_1_ times in both samples (Fig. S7†). This NOE-build-up resembled the observed curve for the short-ranged NOE between protons in the aromatic ring and was therefore determined to correspond to a short-ranged contact to the lipid CH_3_ group. No contacts between the aromatic region of FR and the acyl chain region of the membrane lipids were detected when the experiments were repeated in the gel phase (Fig. S8†). This shows that FR can penetrate deeply into the lipid bilayer only in the liquid-crystalline phase, opening up a way of access to enter the cell and reach its G protein target.

### FR activity in G_q_ proteoliposomes

**Fig. 5:**
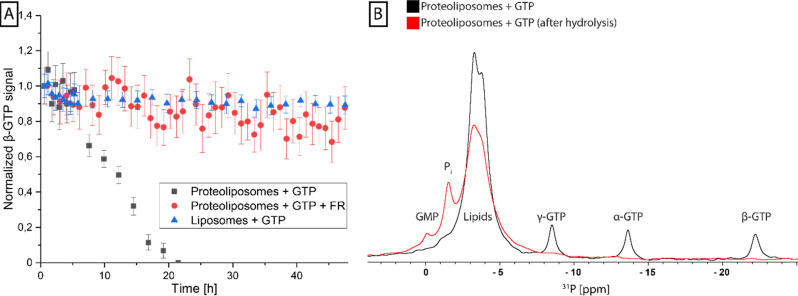
Analysis of GTP hydrolysis of reconstituted G_q_ protein by ssNMR. (A) Several ^31^P-spectra were recorded in a real-time analysis to observe the β-GTP signal in the presence of reconstituted G_q_ protein (gray), FR-inhibited reconstituted G_q_ protein (red) and POPC : POPE : Cholesterol liposomes (blue). The β-GTP signal was fitted using a Lorentzian peak function and the normalized peak integrals were plotted against the experimental time. Error bars correspond to area error of the peak fit. (B) ^31^P-spectra of proteoliposomes after the addition of GTP show three distinct signals corresponding to the α-, β- and γ-phosphates of GTP in addition to the phosphate signals of the lipids (black). After the hydrolysis reaction, only the lipid signal remains and two new resonances of inorganic phosphate (P_i_) and GMP appear (red).

To validate the functionality of the reconstituted G protein, GTP turnover experiments were performed on G_q_ proteoliposomes using time-resolved ^31^P-NMR, which can be used to directly follow changes in GTP, GDP, and free phosphate concentrations over time. Incubation of the proteoliposomes with GTP resulted in a complete consumption of the nucleotide within 20 hours, whereas in the control sample without G protein it was stable over this time range, confirming protein-driven GTP hydrolysis. No GDP build-up could be detected during this period due to the instability of GDP under the experimental conditions, leading to auto-hydrolysis, resulting in GMP-specific resonances. When this experiment was repeated with proteoliposomes that were preincubated with FR, a strong inhibition of GTPase activity could be detected, as FR blocks the exchange of GDP for GTP (Fig. 5).

### Chemical shift changes in G_q_-bound FR

**Fig. 6:**
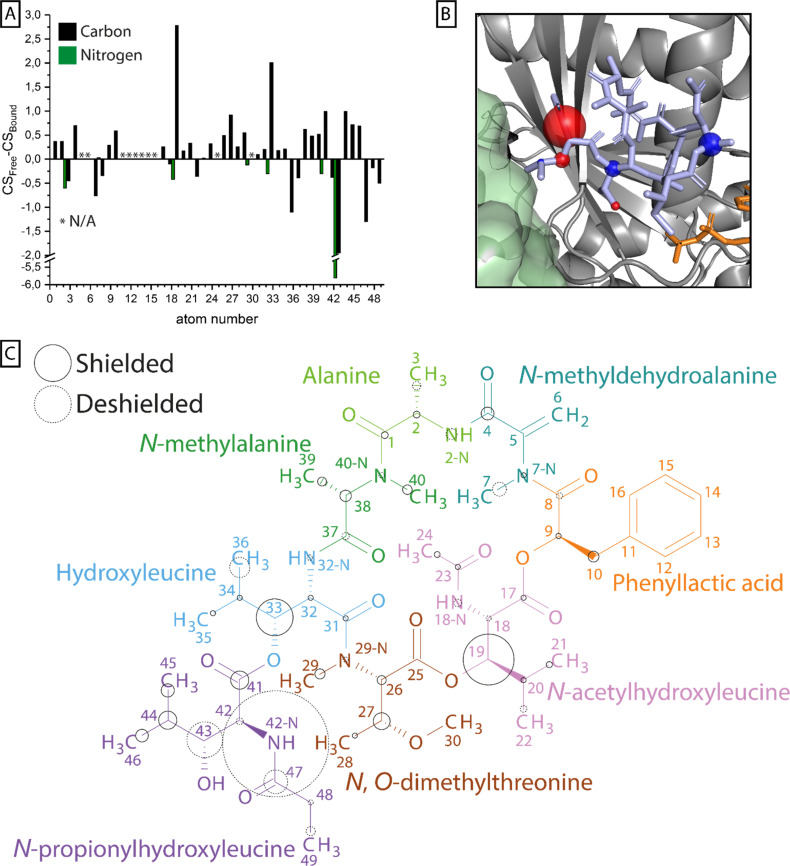
Chemical shift perturbation upon FR binding to G_q_. (A) For all carbon (black) and nitrogen (green) atoms in ^13^C^15^N-labeled FR that could be assigned in solution by L-NMR and in the protein-bound state by ssNMR, the difference in chemical shift is plotted in a bar chart. (B) Nuclei with chemical shift perturbations ≥ 1 ppm are highlighted by spheres and mapped onto the X-ray structure of YM (light blue) in its binding pocket in the Gα subunit (gray) (PDB 3AH8).^23^ The size of the spheres corresponds to the magnitude of the shift, while the color indicates shielding (blue) and deshielding (red). In this structure the Gβ subunit (green) and a bound GDP molecule (orange) are also depicted. (C) The calculated chemical shift perturbation was normalized and mapped onto the FR molecular structure. The size of the circles indicates the magnitude of the shift, while the stroke shows shielding (solid) or deshielding (dashed) of the nucleus upon binding. The coloring scheme of FR was adapted from ref. 45.

To compare the signals of G protein-bound FR with free FR, ^13^C-^13^C correlation, NCA and NCO spectra were recorded. Protonated and methylated nitrogen atoms could be differentiated in these experiments using different ^15^N-CP contact times (Fig. S9 + S10†). The acquired spectra were compared to the L-NMR data in aqueous buffer and the assignment was transferred by assigning ssNMR signals with similar chemical shifts as detected by L-NMR. This was validated by the ^13^C-^13^C-correlation network to neighboring carbons (Fig. S11–S14†) and nitrogen–carbon correlations found in the NCA and NCO spectra. In total 40 of 49 carbons were detected of which 36 could be assigned unambiguously in the bound state as well as all of the nitrogen resonances (Table S1†). Based on these data, chemical shift differences between the free and bound state were calculated and regions of larger perturbations (>1 ppm) were identified (Fig. 6 + Table S1†).

Previous docking studies have shown that except for the *N*-methylalanine and the alanine moieties, modifications in the cyclodepsipeptide inhibitors are not well tolerated in terms of G_q_ binding and inhibitory activity.^24^ CSP analysis can highlight parts of the molecule that undergo changes in their chemical environment upon G_q_ binding, potentially indicating their relevance to the inhibitor's functionality. Those changes can be a result of structural rearrangements, forming or dissolving of interactions like H-bonds, or different solvent accessibility. While resonance assignment of FR in solution was less ambiguous due to strong and narrow signals, ssNMR assignment of the G_q_-bound form in the presence of the membrane environment was more complex. The absence of fast isotropic tumbling of the observed system leads to partial line broadening caused by slow conformational exchange or structural heterogeneity, making the assignment more challenging. Therefore, especially for chemically similar groups like the methyl groups in the isopropyl moieties or the aromatic ring in FR, a reliable analysis of the chemical shift perturbation was not possible. Multiple regions of the inhibitor molecule displayed changes in the chemical shift upon binding, among those most prominently the NH group of the extracyclic *N*-propionylhydroxyleucine moiety (ΔCS = 5.81 ppm). Noticeable chemical shift perturbations (ΔCS ≥ 1 ppm) were also found in other parts of the *N*-propionylhydroxyleucine moiety as well as in the isopropyl groups of *N*-acetylhydroxyleucine and hydroxyleucine. The large cluster of chemical shift perturbation in the *N*-propionylhydroxyleucine moiety of FR fits the current binding model in which interactions are formed between the cyclodepsipeptide inhibitor and residues of switch I in G_q_ like hydrogen bonds between E191 and the free hydroxyl/amide group and I189, which is part of the hydrophobic interaction network that coordinates the isopropyl group in this anchor 1 region of the inhibitor molecule.^23^ Formation of a hydrogen bond also explains the large CSP observed for 42-NH, since it has been shown that an increase in N–H bond length leads to deshielding of the nitrogen nucleus.^46^ The methyl groups of the three isopropyl moieties in the inhibitor molecules displayed different chemical shifts in solution, indicating no completely free and fast rotation of those groups even in the unbound state (Table S1†). Once bound to the G protein, only the methyl groups of hydroxyleucine could be assigned unambiguously due to their large difference in chemical shift, which even increased upon binding. The latter observation fits the proposed hydrophobic interactions of this group with I190 and T187 and also explains the large CSP observed for 33-C.^23^ The last notable CSP in *N*-acetylhydroxyleucine fits the previously proposed relevance of this FR-specific lipophilic anchor 2 region, leading to a longer residence time compared to YM.^17^ The *N*-methylalanine and the alanine moieties have not been identified as part of the pharmacophore of the inhibitor molecule and accordingly no large CSPs were found for those residues.^24^

Earlier studies have found that cyclodepsipeptide inhibitors occur in different isomeric forms, with the ratio depending on the solvent and varying between FR and YM.^25,47^ The difference between the isomers lies in the conformation of the various amide groups in the inhibitor molecule. While in the X-ray structure (PDB: 3AH8)^23^ the G protein-bound YM displays a *trans* amide bond between *N*-methyldehydroalanine and phenyllactic acid (*trans* YM), the predominant conformation of both FR and YM in water features a *cis* amide bond at this position (*cis* FR/YM). By comparing published chemical shifts of the isomers, we identified four carbon resonances that displayed a large sensitivity towards the *cis*/*trans* conformation (CSP ∼ 3–10 ppm).^48^ Of those resonances, two (26, 29) are close to the amide bond between *N*, *O*-dimethylthreonine and hydroxyleucine and one (40) is close to the amide bond between *N*-methylalanine and alanine. Both amide bonds are in different conformations in *cis*/*trans* YM, respectively. We find that for all three resonances, which show a high sensitivity towards the isomer conformation and are close to amide bonds, our detected chemical shifts correspond to the *cis* isomer. This indicates that FR is not only mainly in the *cis* conformation in solution but also when it is bound to its G protein target. This observation aligns with published molecular dynamics simulations.^25^ These simulations confirmed that the *cis* YM structure, the major isomer in aqueous solution, also fits the electron density map of the bound YM in the X-ray structure and displays a higher affinity for the binding site compared to the *trans* YM.^25^

### Dynamics of bound FR

**Fig. 7:**
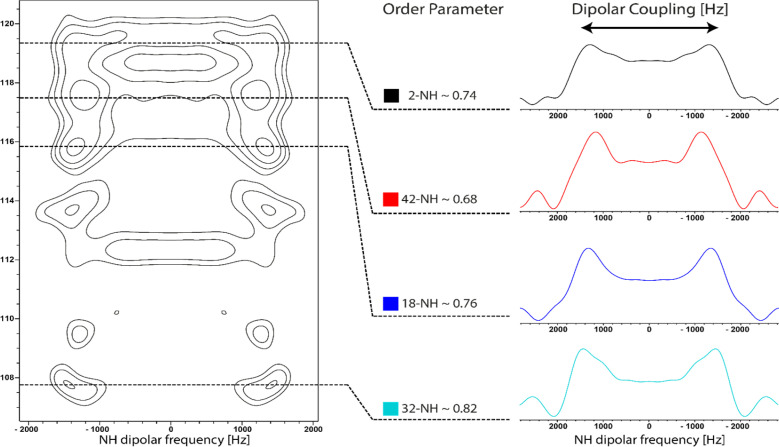
Analysis of inhibitor NH dynamics by ssNMR. 2D ^15^N-SLF spectrum of ^13^C^15^N-FR bound to reconstituted G_q_ protein. Slices corresponding to the chemical shift of the four amide nitrogens in FR were isolated and dipolar order parameters were calculated. Based on the accuracy of the determination of the dipolar splitting an error of ± 0.02 is estimated for the calculated order parameters.

Dynamics of the inhibitor bound to the protein were assessed with a separated local field (SLF) experiment to determine dipolar coupling parameters of the protonated nitrogens, which are well dispersed in the molecule. 1D Slices were taken from the 2D spectra according to the previously determined expected chemical shift of the amides. The signal splitting revealed similar order parameters for amides 2-NH and 18-NH of 0.74 and 0.76 respectively. For amide 32-NH, a slightly higher order parameter of 0.82 was measured. Additionally, at the chemical shift of 42-NH, a slightly more mobile population was detected with an order parameter of 0.68. Analysis of these values must take into account the additional signals in the SLF-experiment that do not correspond to the expected chemical shift regions, due to G_q_-binding impurities in the sample. Because of this, no detailed analysis can be conducted, however, it can be concluded that FR is bound to G_q_ in a relatively rigid binding mode. All regions of the cyclodepsipeptide exhibit similar dynamics. The observed lower order parameter of 42-NH in the *N*-propionylhydroxyleucine moiety fits the previously observed large CSP for this nucleus. Since the N–H bond length increases upon formation of an hydrogen bond, the NH dipolar coupling decreases, leading to reduced splitting in the SLF spectrum.^49^ Moreover, the 32-NH amide in the hydroxyleucine moiety has been identified as being the most rigid part of the structure (Fig. 7). In comparison, the measurement of μs/ms-dynamics relaxation parameters of free tumbling FR in solution showed reduced T_2_ values for 2-NH and 32-NH, hinting at reduced mobilty of the amides that are part of the cyclic backbone of FR compared to the side chain amides 18-NH and 42-NH. Additionally, relaxation dispersion experiments detected higher rates of chemical exchange for 2-NH and to a lesser degree 32-NH, showing that those parts of the inhibitor sample different chemical environments. Measurement of the heteronuclear NOE displayed high mobility for all NH groups in the range of ps-dynamics (Table S2†).

### Spatially resolved conformational response of the G protein

Multiple regions of the protein are either directly involved in binding of cyclodepsipeptide inhibitors, as shown by structural data on bound YM,^23^ or show an indirect response to binding of the inhibitor, as proposed by MD simulations.^13,25^ To investigate the conformational response of the G protein to inhibitor binding, structural alterations of the G protein itself must be detected. This requires isotope labeling, which is challenging for the G_q_ protein due to the necessity for expression in insect cells. Instead, the FR-sensitive Gα_i_ protein mutant Gα_i/q_, which can be expressed and isotope labeled in *E. coli*, was chosen as a model.^21^ The gene for Gα_i/q_ was subcloned from an insect cell expression plasmid (kindly provided by the Schertler group, PSI Villigen) into the pET16b vector for *E. coli* expression. After purification, biochemical analysis confirmed that the protein was homogeneous and pure, with a yield suitable for NMR experiments (Fig. S15†). Notably, the yield did not decrease upon isotope labeling. The chimeric Gα_i/q_ protein was further characterized by radioligand binding studies using the FR-derived [^3^H]PSB-15900 as a radioligand.^14^ The soluble recombinant protein bearing a 10x histidine tag was attached to metal affinity chromatography beads, which allowed to perform filtration assays to separate bound from unbound radioligand. Competition binding studies with increasing concentrations of FR were performed resulting in displacement of the radioligand (Fig. S16†). A *K*_D_ value of 0.271 ± 0.069 μM was estimated for FR and a *B*_max_ value of 36.3 ± 8.8 pmol mg^−1^ of protein was calculated. These results clearly show that the employed chimeric Gα_i/q_ protein is properly folded and binds FR with high affinity.

**Fig. 8:**
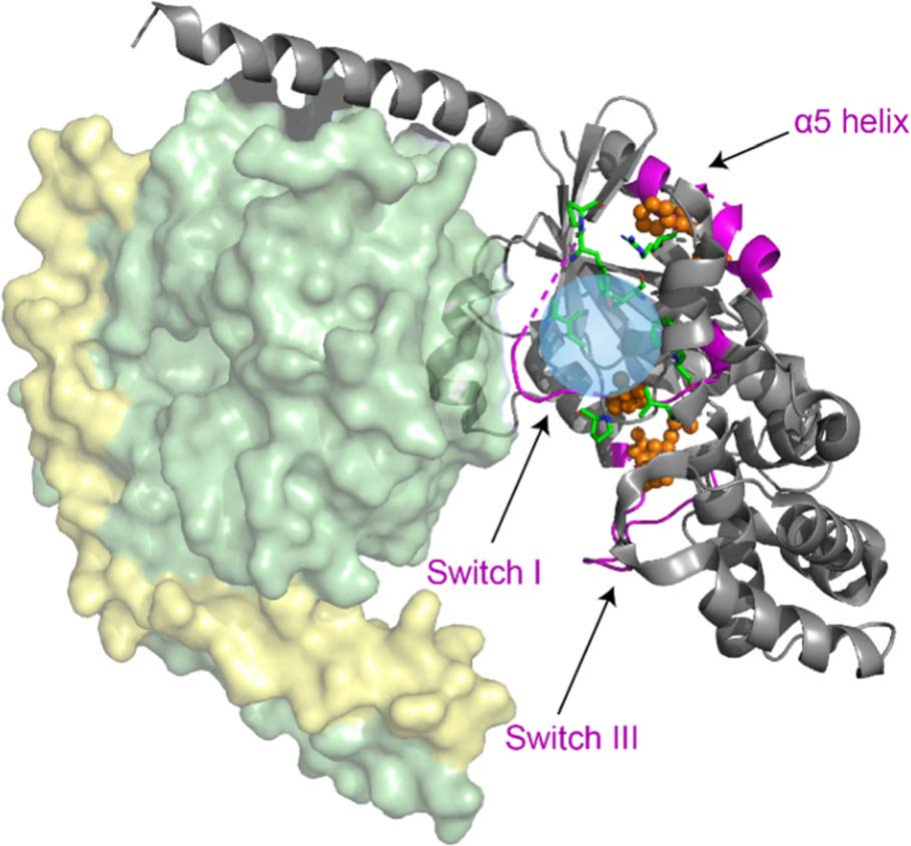
Structural model of the G_i/q_ heterotrimer (modified from PDB: 1GP2). Eight mutations (green) were introduced into the Gα_i_ protein to generate FR-binding Gα_i/q_. Probed structural motifs (purple) and the detected unique pairs (orange spheres) are highlighted. The blue sphere marks the inhibitor binding site. DNP-enhanced ssNMR experiments were conducted on the isolated Gα_i/q_ subunit.

**Fig. 9:**
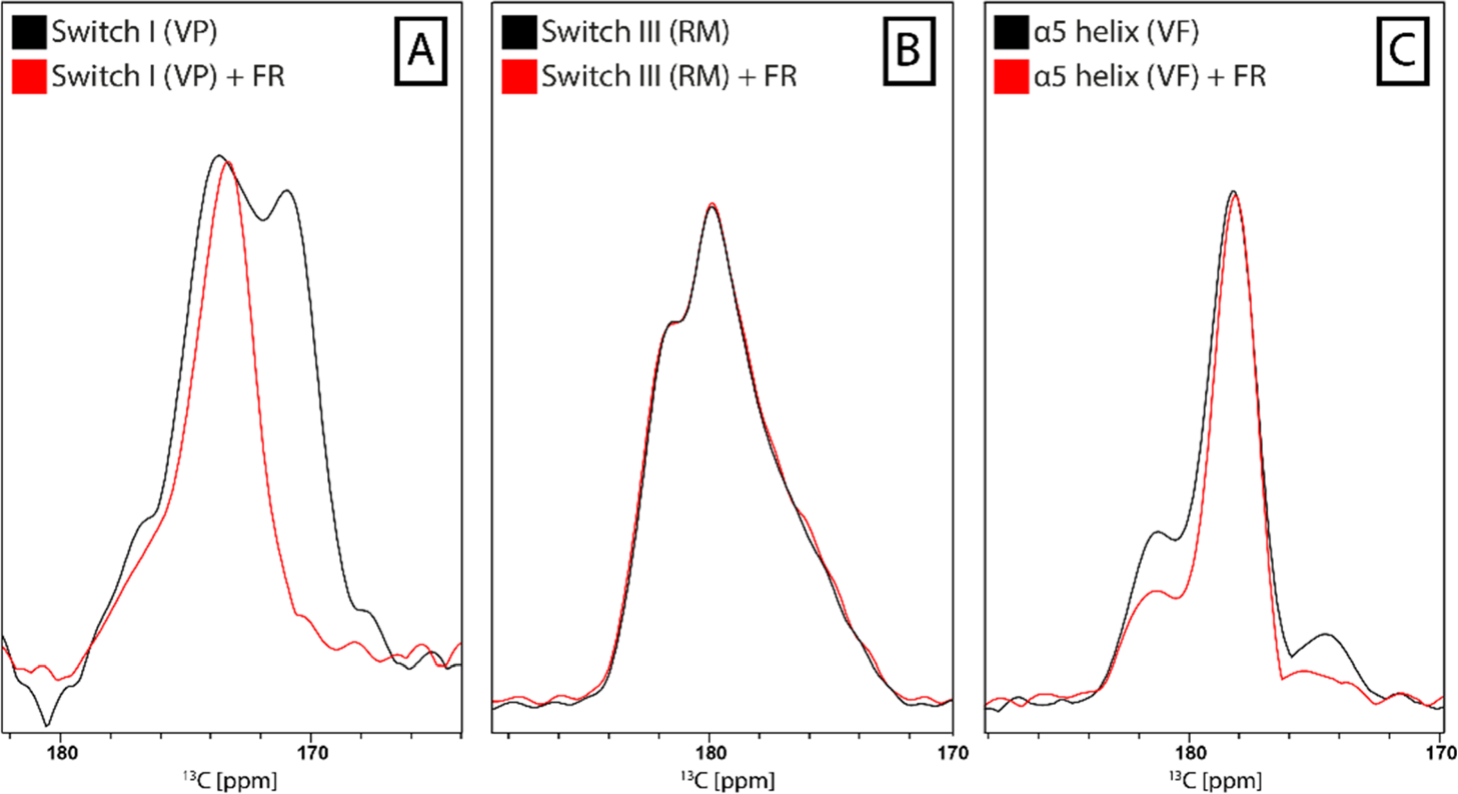
Spatially resolved protein response to FR binding by DNP-enhanced ssNMR. ^13^C-NCOCX spectrum of frozen Gα_i/q_ solution (black). Labeling schemes were chosen to detect one unique pair signal in switch I (A), switch III (B) or in the α5 helix (C). Addition of FR to the samples leads to narrowing of the signal in switch I, no effects in switch III and changes in the intensity of the flanking signals in the α5 helix (red).

In order to collect information about conformational changes in a spatially resolved manner, a unique pair labeling approach was chosen,^50^ resulting in signals in the NMR spectra from a unique pair of neighboring amino acids in NCO type experiments. We selected the V179-P180 pair in switch I, the R242-M243 pair in switch III, and the V335-F336 pair in the α5 helix, all of which contain amino acids not affected by metabolic scrambling in *E. coli*. The VP pair is directly involved in FR binding, whereas the RM and VF pair are more distant but predicted by MD simulations to respond to inhibitor binding (Fig. 8).^13,25^ To obtain sufficient signal intensity in the experiments, spectra were recorded using DNP-enhanced ssNMR. This hybrid electron paramagnetic resonance (EPR)-ssNMR method can enhance sensitivity by orders of magnitude. It requires sample doping with polarizing agents and data acquisition under cryogenic conditions. Under those conditions, the full sampled conformational space is conserved and reflected in NMR line shapes, which can then be analyzed. All measured samples displayed a signal enhancement in the range of 60–80-fold. By comparing the NCO peak position and line shape, local changes can be observed between the free and inhibitor-bound G protein. The experiment showed a clear narrowing of the ^13^C valine signals in the NCOCX spectrum of the VP-pair sample upon binding of both FR and YM. The VF-pair in the α5 helix of Gα_i/q_ also showed a response to FR-binding, evidenced by the decrease of the two peaks flanking the main signal. For the RM-pair in switch III, no big changes were detectable (Fig. 9 + S17†). Consequently, the switch I region, which is directly involved in inhibitor binding, seems to show a pronounced response to the addition of cyclodepsipeptide inhibitors. Additionally, the more distant region of the α5 helix, despite having no direct contact with the inhibitor binding site, shows a structural response to FR binding, as suggested by the previously published MD simulation data. However, the proposed effect on the switch III region could not be confirmed.

**Fig. 10:**
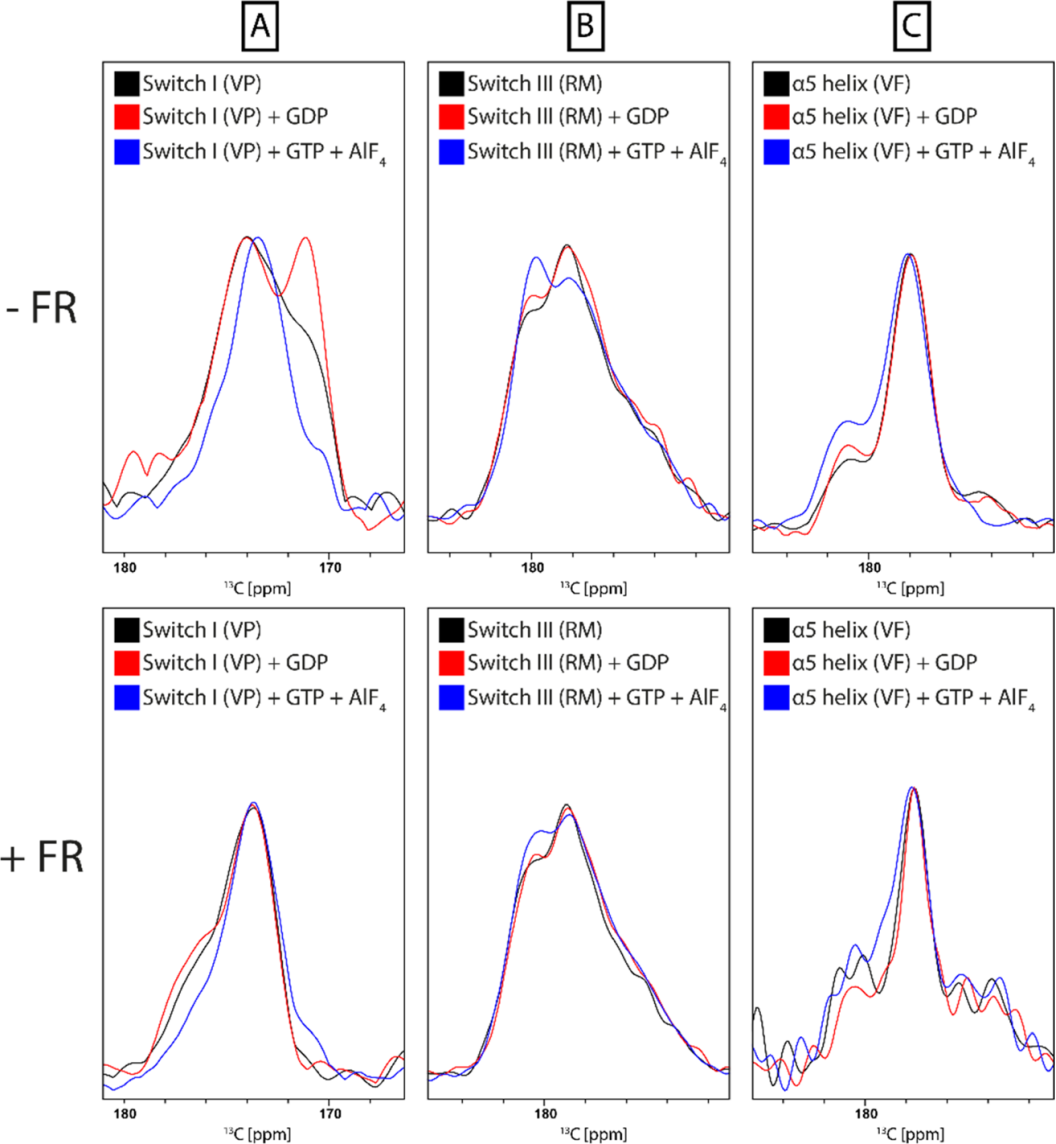
Spatially resolved protein response to nucleotide binding by DNP-enhanced ssNMR. ^13^C-NCOCX spectrum of frozen Gα_i/q_ solution (black). Labeling schemes were chosen to detect one unique pair signal in switch I (A), switch III (B) or in the α5 helix (C). Signal line shapes are changing for switch I and switch III upon addition of GDP (red) or trapping of the protein in the GTP-bound state by addition of GTP and AlF_4_ (blue). Only minor changes are observed in the α5 helix. Line shape deviations from the state without nucleotide addition (black) that are visible without prior FR incubation (top) are attenuated upon prior protein incubation with FR (bottom).

The same set of samples was also used to study the impact of nucleotide binding on the conformation of the protein and to assess how this is affected by prior inhibition with FR. The switch I and II regions in guanine nucleotide-binding proteins generally and additionally switch III in Gα proteins are sensitive towards the bound nucleotide and undergo structural changes upon the release of the γ-phosphate during GTP-hydrolysis.^51,52^ It has been shown previously that the structural dynamics of the Gα subunit is dependent on the presence and type of nucleotide bound to the Ras domain.^53,54^ While the apo and GDP-bound protein are more flexible, the GTP-bound G protein adopts a more rigid conformation. For each sample spectra were recorded under the following conditions: Gα_i/q_, Gα_i/q_ + GDP, Gα_i/q_ + GDP + GTP/AlF_4_, Gα_i/q_ + FR, Gα_i/q_ + FR + GDP, Gα_i/q_ + FR + GDP + GTP/AlF_4_. Addition of AlF_4_ occupies the binding site of the third phosphate group of GTP in the G protein and thereby mimics GTP binding.^55^ Line shape analysis of the NCO signal revealed structural changes between the initial state after purification, which should be at least partially nucleotide-free, GDP-bound, and GTP-bound state in the switch I and III regions but only minor changes for the α5 helix. This was expected since the switch regions in G proteins are the main conformational responders to nucleotide binding.^56^ Comparison of the observed changes with samples that were preincubated with FR showed a strong suppression of those effects due to the presence of the inhibitor. This confirms its mode of action, which involves keeping the protein locked in the inactive GDP-bound state (Fig. 10).

## Conclusions

The class of cyclodepsipeptide inhibitors provides a powerful tool for studying GPCR-G protein signaling due to their mode of selective inhibition of nucleotide exchange in Gα_q_. This inhibitory mechanism can be transferred by mutagenesis to other G proteins, making this instrument even more versatile.^21,26,27^ Purification of the inhibitor FR900359 in a ^13^C^15^N isotope-labeled form enabled the characterization of this cyclodepsipeptide in a membrane environment and also directly bound to its G protein target. Here, we present an extensive resonance assignment of FR in aqueous solution and bound to the membrane-anchored G_q_ protein heterotrimer. Chemical shift perturbation analysis between those two states, which both mimic native-like states, revealed parts of the inhibitor molecule that are particularly affected by protein binding. This is in line with previous docking studies, kinetic and structural data.^17,23,24^ Our findings show that FR is in the *cis* conformation in both the solubilized as well as in the G_q_-bound state. This supports previous MD simulations proposing a higher binding affinity for the *cis* compared to the *trans* isomer found in the G protein-bound X-ray structure of YM.^25^ In a recently solved X-ray structure of FR in complex with G_11_ the *cis* isomer was also observed and a contact between Gβ and the *N*-propionylhydroxyleucine moiety of the inhibitor was identified.^57^ The large CSP observed for 42-NH indicates a FR-G_q_ hydrogen bond which is compatible with the heterotrimer-stabilizing hydrogen bond network between FR and G_q_ as derived from the before mentioned X-ray structure. Although FR does not interact with our liposomes in the gel phase, bound FR was detected by cross-polarization experiments in the physiologically relevant liquid-crystalline phase. Further investigation of intramolecular and intermolecular FR-lipid contacts by ^1^H-^1^H-NOESY experiments revealed full penetration of membranes by FR in the more fluid liquid-crystalline phase, providing a mode of access for the small molecule into the cell. The dynamics of the FR molecule in the bound state were studied by SLF-experiments on the well dispersed nitrogen atoms in the inhibitor molecule, showing an overall rigid FR structure in the bound state. The weaker dipolar coupling for the N–H bond in the *N*-propionylhydroxyleucine moiety again points to the formation of a hydrogen bond network in this part of the inhibitor binding pocket, possibly involving the Gβ subunit.^57^ On the protein side of the system, we observed the hydrolysis activity of the membrane-anchored G_q_ protein in real-time by ^31^P-NMR, as well as the inhibitory effects of FR on the hydrolysis reaction due to the diminished nucleotide exchange. For a spatially-resolved analysis of structural changes in the G protein upon inhibitor binding, we utilized the aforementioned sensitization of otherwise FR-insensitive G protein families. Gα_i/q_, a FR-sensitive mutant of Gα_i_, displayed high affinity binding of FR in a radioligand binding assay and the system was analyzed by DNP-enhanced ssNMR. Based on the chosen unique pair labeling scheme, the line shape analysis of the signals revealed spatially resolved responses of the protein. We could demonstrate an allosteric response in the α5-helix in addition to the expected effect in the direct binding site (switch I). This finding supports previous MD simulations^13,25^ but a predicted response within the switch III region was not observed. Additionally, the effects of nucleotide binding on the switch regions were observed as changes of the conformational space of those structural motifs upon switching between the GDP- or GTP-bound state. In line with the cyclodepsipeptide's mode of action as a GDI, these observed changes could be suppressed by prior incubation of the protein with FR. In summary, L-NMR, ssNMR, as well as DNP-enhanced experiments allowed for the acquisition of a broad range of diverse information about the FR-G protein system. In conjunction with other structural and biochemical data, these methods help assess the current model of the FR binding mode. Especially, the native-like sample conditions, like inclusion of the membrane and an aqueous environment, that can be combined with NMR, make this method a valuable lens through which one can study such systems.

## Data availability

NMR raw data can be obtained *via* the Goethe University data repository (https://gude.uni-frankfurt.de/).

## Author contributions

CB prepared samples, carried out solid-state NMR experiments, performed data analysis, interpreted data, prepared figures and wrote the paper. WH and GK produced labeled FR, analyzed samples and contributed to figures and paper writing. JM and GS provided plasmids and initial protocols and contributed to discussions and experimental design. FL carried out solution-state NMR experiments and performed data analysis. JB helped in performing solid-state NMR experiments, helped with data interpretation and discussion and contributed to the project design. JN and CM provided the radioligand binding studies and contributed to paper writing. DH provided G_q_, helped with data interpretation and contributed to paper writing. CG designed the project, contributed to experimental design, data interpretation, discussion and paper writing.

## Conflicts of interest

There are no conflicts to declare.

## Supplementary Material

SC-015-D4SC01950D-s001
