# Supplementary material for: Structural response of G protein binding to the cyclodepsipeptide inhibitor FR900359 probed by NMR spectroscopy
Source: Chem Sci. 2024 Jul 4;15(32):12939–56. doi: 10.1039/d4sc01950d (PMC11323312; doi:10.1039/d4sc01950d)
Supplement: SC-015-D4SC01950D-s001 [file SC-015-D4SC01950D-s001.pdf]

Electronic Supplementary Material (ESI) for Chemical Science.
This journal is © The Royal Society of Chemistry 2024

ARTICLE

Supporting Information

Received 00th January 20xx,
Accepted 00th January 20xx

DOI: 10.1039/x0xx00000x

Structural response of G protein binding to the cyclodepsipeptide inhibitor FR900359 probed by NMR spectroscopy

Christian Bonifer,[a] Wiebke Hanke,[b] Jonas Mühle,[c] Frank Löhr,[a] Johanna Becker-Baldus,[a] Jessica Nagel,[d] Gebhardt Schertler,[c] Christa E. Müller,[d] Gabriele M. König,[b] Daniel Hilger,*[e] and Clemens Glaubitz*[a]

(A) Figures

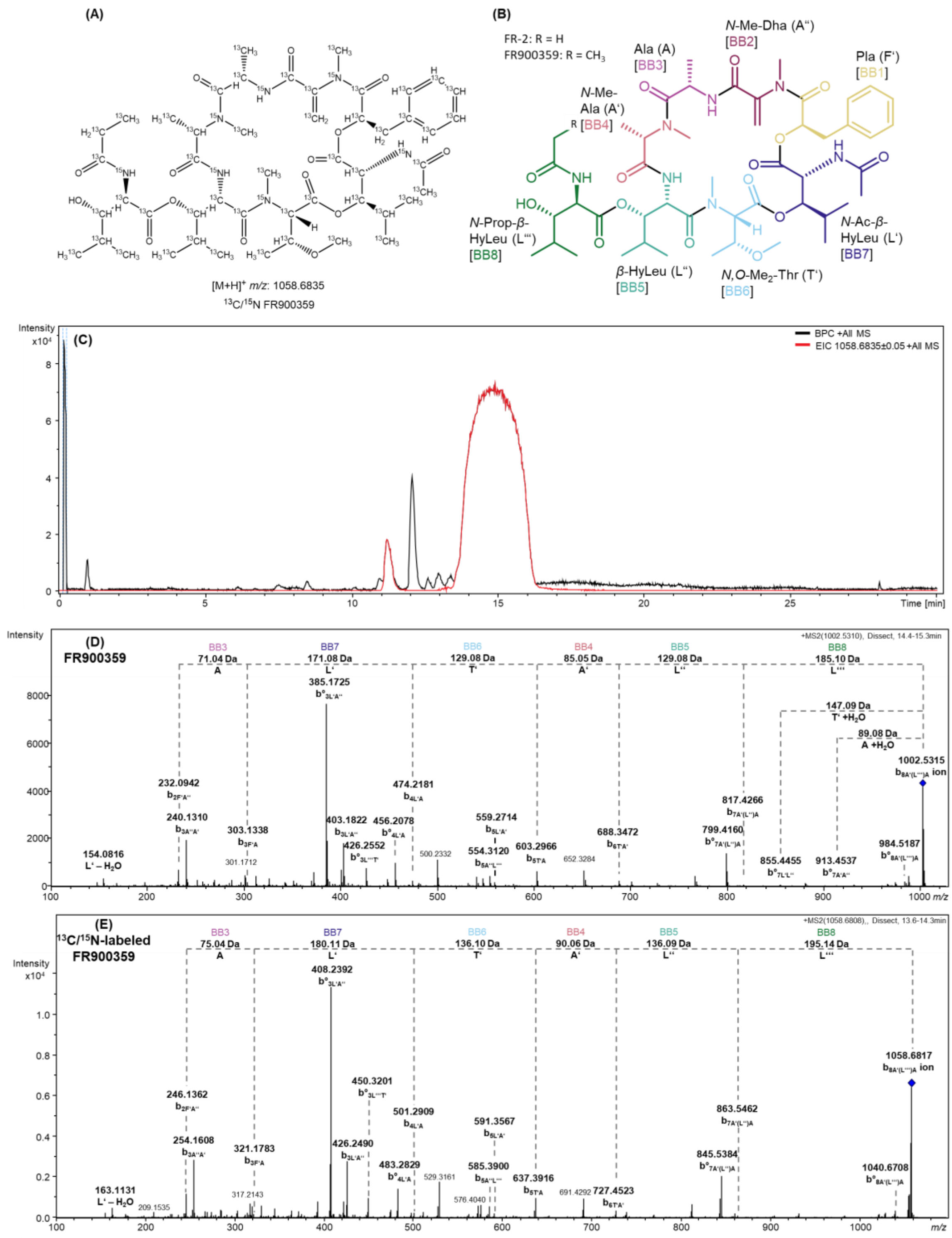

Figure S1: (A) Structure and exact mass of the proton adduct of the uniformly $^{13}C^{15}N$-labeled FR. **(B)** Structure of the unlabeled FR and FR-2 with colored and named building blocks. **(C)** LC-MS/MS baseline peak chromatogram (BPC) of the FR fraction collected after 60 min and extracted ion chromatogram (EIC) for the *m/z* of the uniformly labeled FR. Fragmentation pattern of FR **(D)** and uniformly $^{13}C^{15}N$-labeled FR **(E)** with fragments labeled following the nomenclature by Ngoka [1] based on Biemanns moficiations [3] of Roepstorffs nomenclature in one-letter amino acid code [4]. b° = b-ion with loss of water. L' = *N*-Ac-β-HyLeu, A = Ala, A' = *N*-Me-Ala, T' = *N*,*O*-Me_2-Thr, L''' = *N*-Prop-β-HyLeu, L'' = β-HyLeu, F' = Pla, A'' = *N*-Me-Dha.

 This journal is © The Royal Society of Chemistry 20xx

Please do not adjust margins

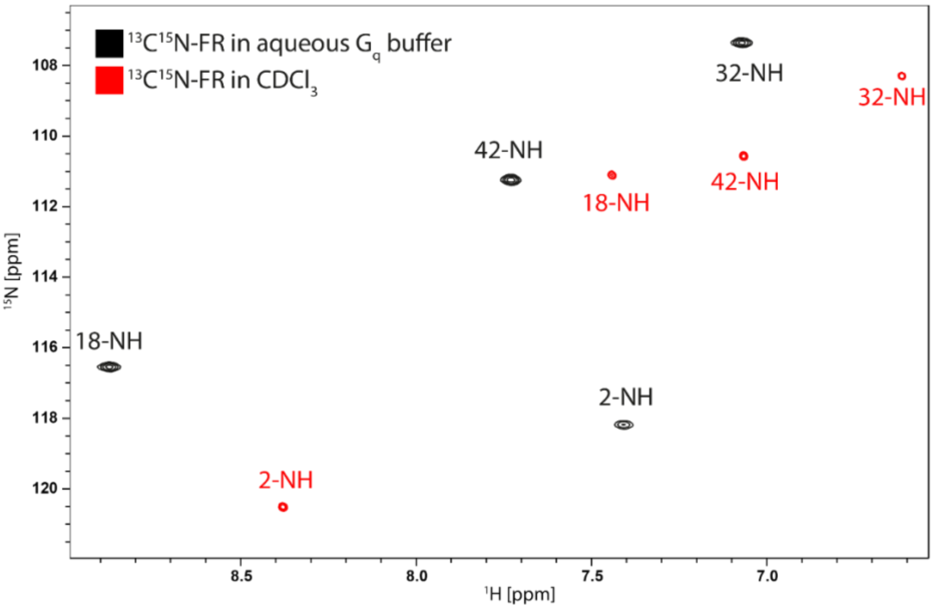

Figure S2: Solvent dependence of inhibitor signals by L-NMR. ^{15}N-^{1}H-BEST-TROSY spectrum of $^{13}C^{15}N$-labeled FR dissolved in aqueous G_q buffer (black) compared to $CDCl_3$ (red). The detected signals show strong changes in chemical shift between the two solvents.

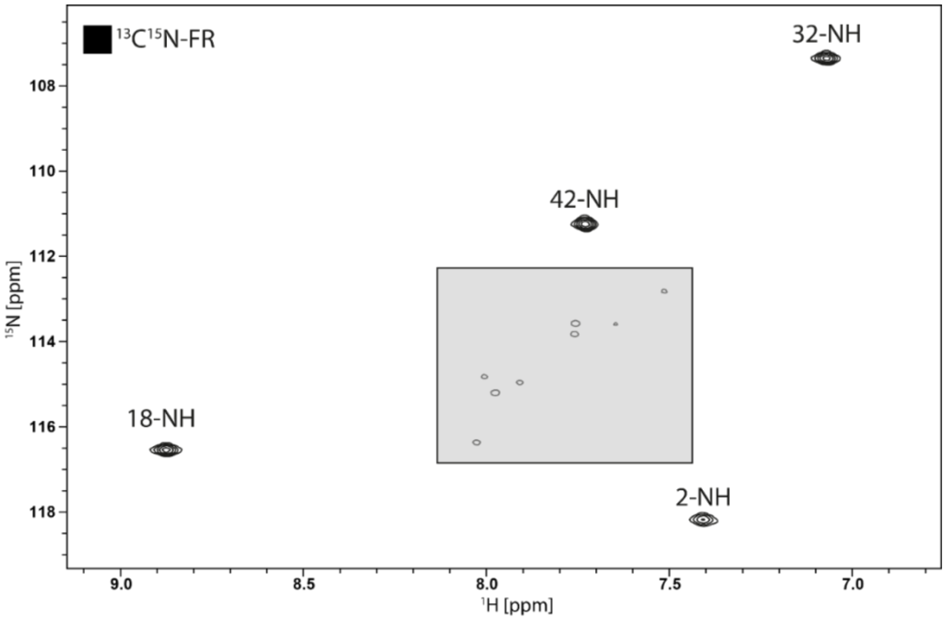

Figure S3: Inhibitor sample impurities detected by L-NMR. ^{15}N-^{1}H-BEST-TROSY spectrum of $^{13}C^{15}N$-labeled FR in aqueous buffer solution shows four strong peaks that can be assigned to the protonated nitrogen atoms in the molecule. Additional weaker signals, corresponding to labeled impurities in the sample, were also detected (gray box).

Please do not adjust margins

Please do not adjust margins

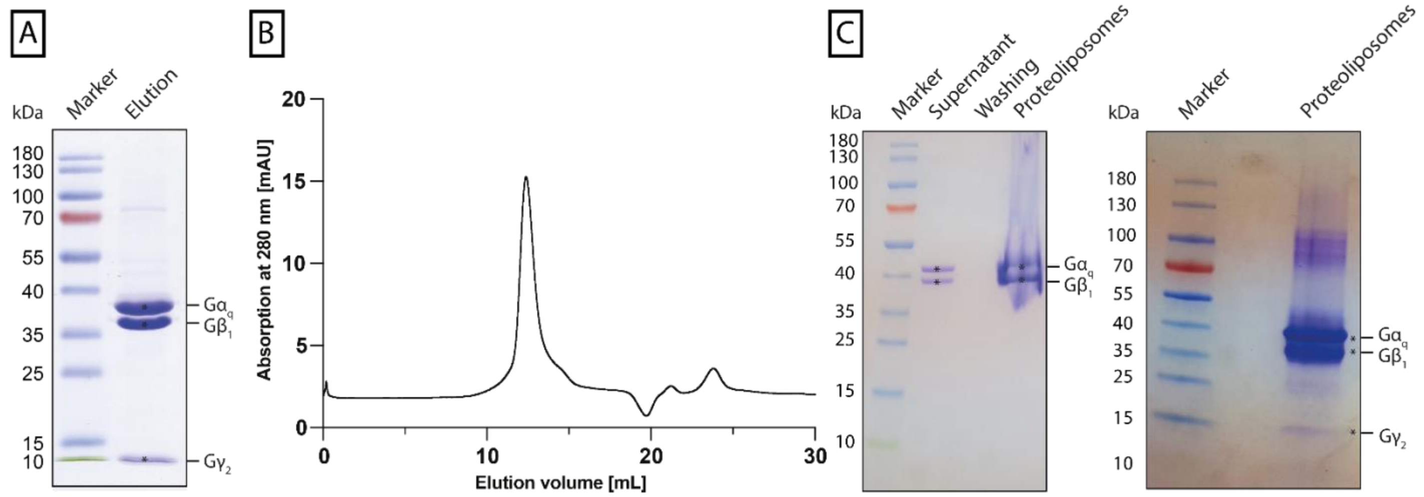

Figure S4: Biochemical characterization of G_q protein expression and reconstitution. **(A)** SDS-PAGE analysis of the heterotrimeric G_q protein, the bands for the three subunits are highlighted. **(B)** Analytical gel filtration analysis displays a distinct signal corresponding to the heterotrimer. **(C)** After reconstitution of the heterotrimeric G protein into POPC:POPE:Cholesterol liposomes, the proteoliposomes were pelleted, resuspended in lipid buffer for washing and pelleted again for NMR rotor packing. Analysis of the supernatant of the first (supernatant) and second (washing) centrifugation, as well as the proteoliposome pellet was conducted by SDS-PAGE. Two bands can be detected at ~ 40 kDa for the supernatant and proteoliposome samples, corresponding to reconstituted $G\alpha_q$ and $G\beta_1$. The G_γ subunit was visualized on a separate gel with a higher gradient.

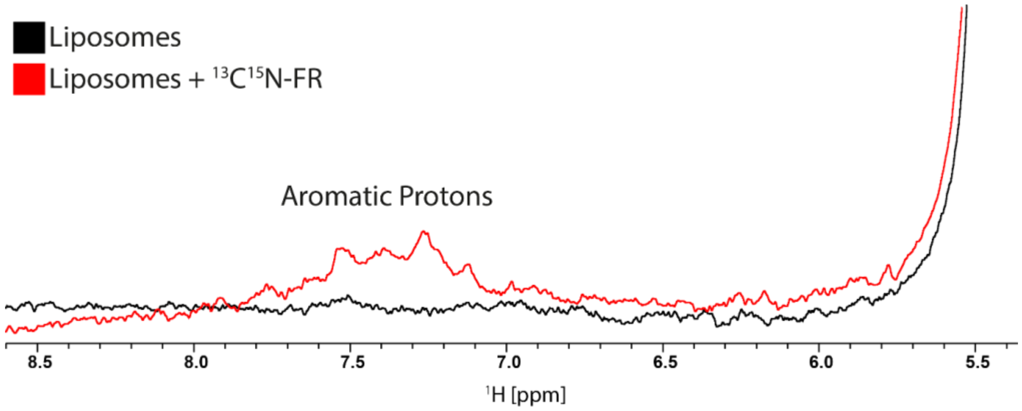

Figure S5: Region of aromatic protons in ^{1}H-NMR. Signals for aromatic protons between 7 and 8 ppm are visible only in the liposome sample in the liquid-crystalline phase upon addition of FR (red). Those signals correspond to the phenyllactic acid moiety of FR, while no such protons are found in POPC:POPE:Cholesterol liposomes (black).

This journal is © The Royal Society of Chemistry 20xx

Please do not adjust margins

Please do not adjust margins

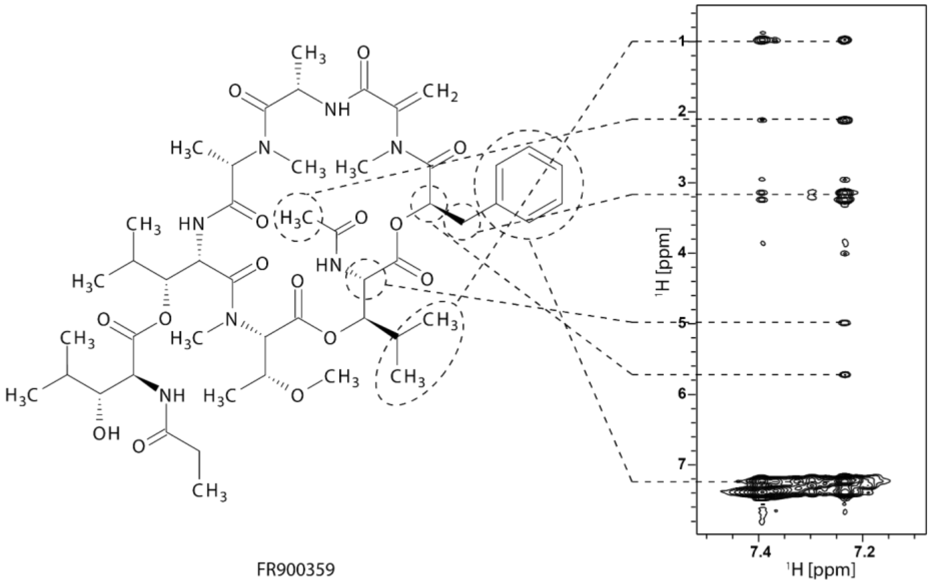

Figure S6: Assignment of L-NMR NOESY spectrum of FR in solution. The aromatic region of the ^{1}H-^{1}H-NOESY (400 ms mixing time) shows cross-peaks that represent intramolecular contacts of the aromatic ring of the phenyllactic acid moiety of FR to other parts of the inhibitor molecule. The dotted lines indicate chemical groups that correspond to the respective NOESY signals.

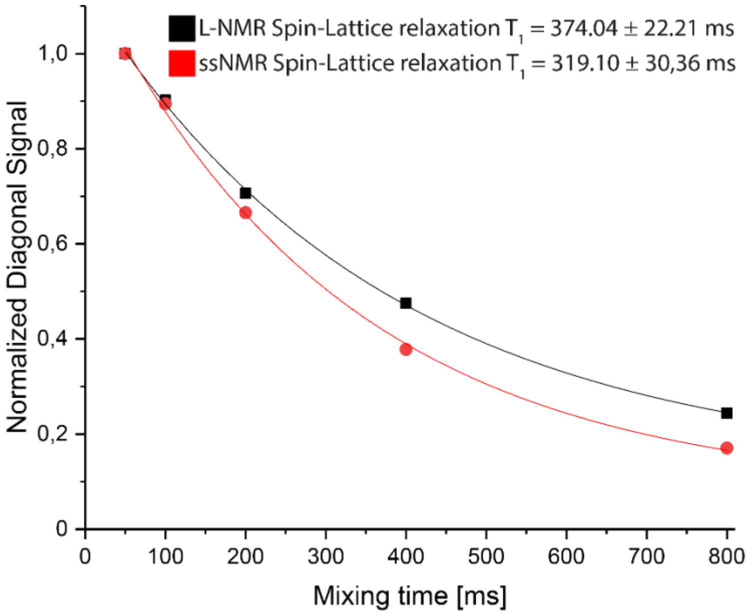

Figure S7: Determination of the spin-lattice relaxation rate T_1 of the NOESY samples. By plotting the intensities of the diagonal signals of the aromatic ring of FR in solution (black) or incubated with POPC:POPE:Cholesterol liposomes in the liquid-crystalline phase (red), the exponential decay of the signal could be fitted to yield the rate of T_1-relaxation for the samples.

This journal is © The Royal Society of Chemistry 20xx

Please do not adjust margins

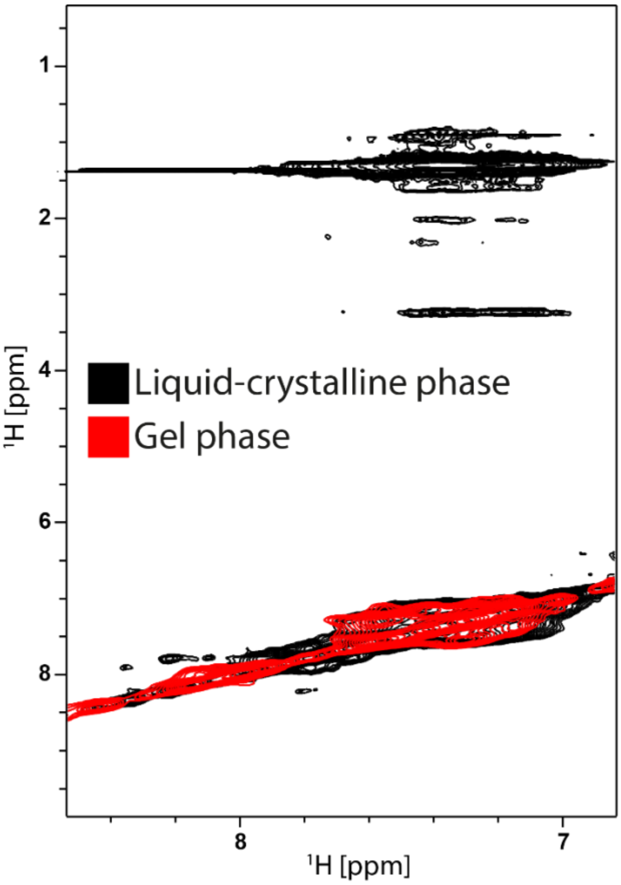

Figure S8: Influence of the membrane phase on FR-lipid contacts. The aromatic region of the ssNMR ^{1}H-^{1}H-NOESY (200 ms mixing time) shows intermolecular contacts between FR and the POPC lipid only when the sample is in the liquid-crystalline phase but not for the gel phase.

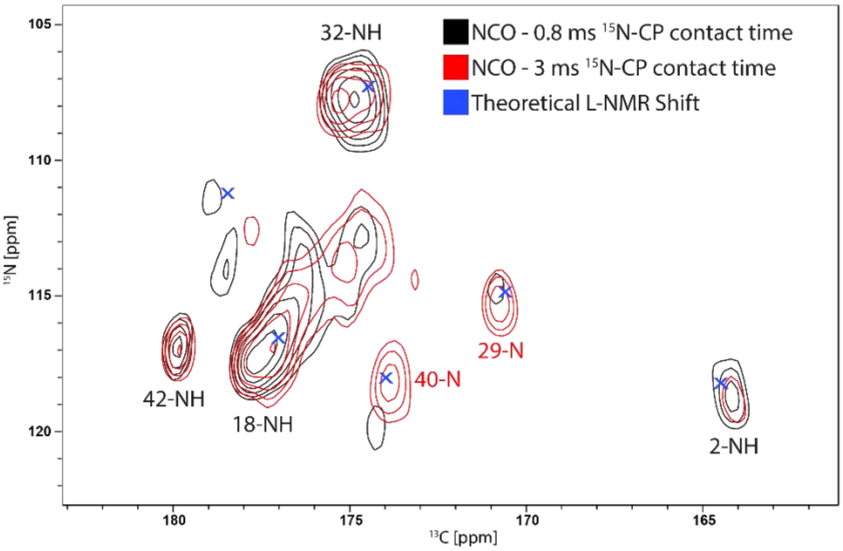

Figure S9: NCO spectra of ^{13}C^{15}N-labeled FR bound to the membrane-anchored G_q heterotrimer. Additional resonances, corresponding to methylated nitrogen atoms, are appearing in the spectrum when longer ^{15}N-CP contact times are applied. The theoretical L-NMR chemical shift of the annotated signals is marked by blue crosses.

 This journal is © The Royal Society of Chemistry 20xx

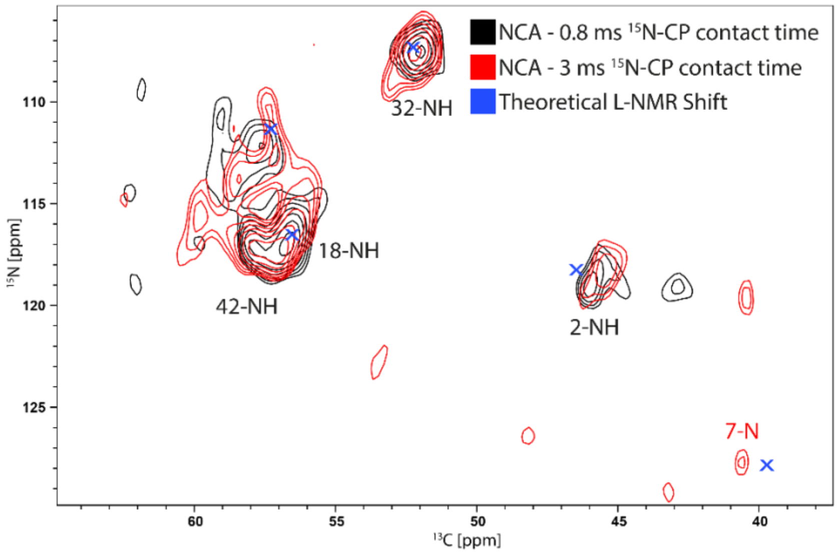

Figure S10: NCA spectra of $^{13}C^{15}N$-labeled FR bound to the membrane-anchored G_q heterotrimer. An additional resonance, corresponding to one methylated nitrogen atom, is appearing in the spectrum when longer ^{15}N-CP contact times are applied. The theoretical L-NMR chemical shift of the annotated signals is marked by blue crosses.

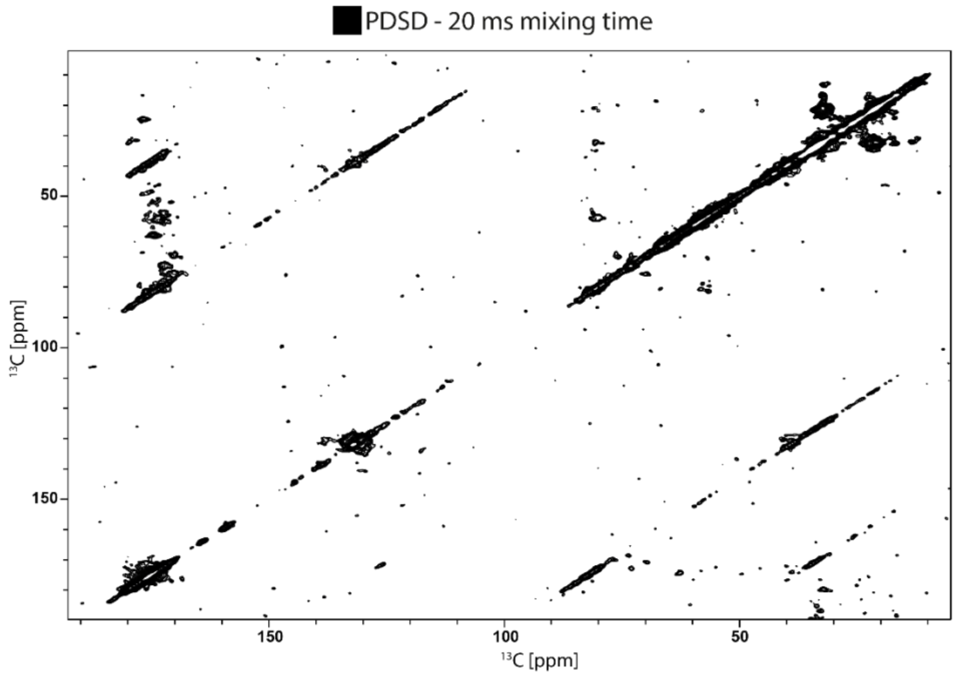

Figure S11: $^{13}C^{13}C$-PDSD spectrum of $^{13}C^{15}N$-labeled FR bound to the membrane-anchored G_q heterotrimer with 20 ms mixing time.

Please do not adjust margins

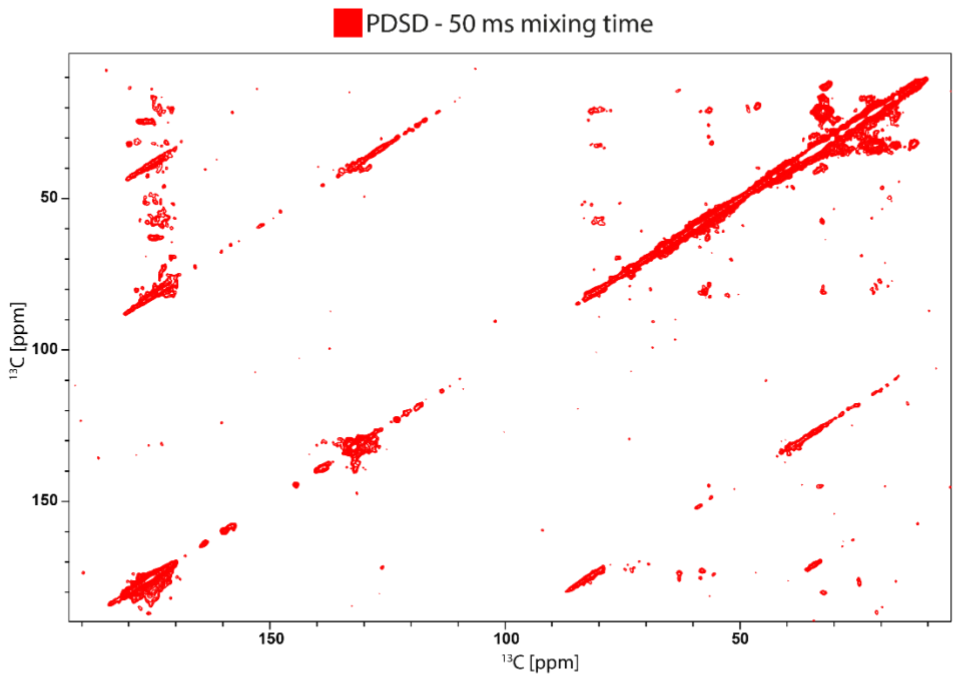

Figure S12: $^{13}C^{13}C$-PDSD spectrum of $^{13}C^{15}N$-labeled FR bound to the membrane-anchored G_q heterotrimer with 50 ms mixing time.

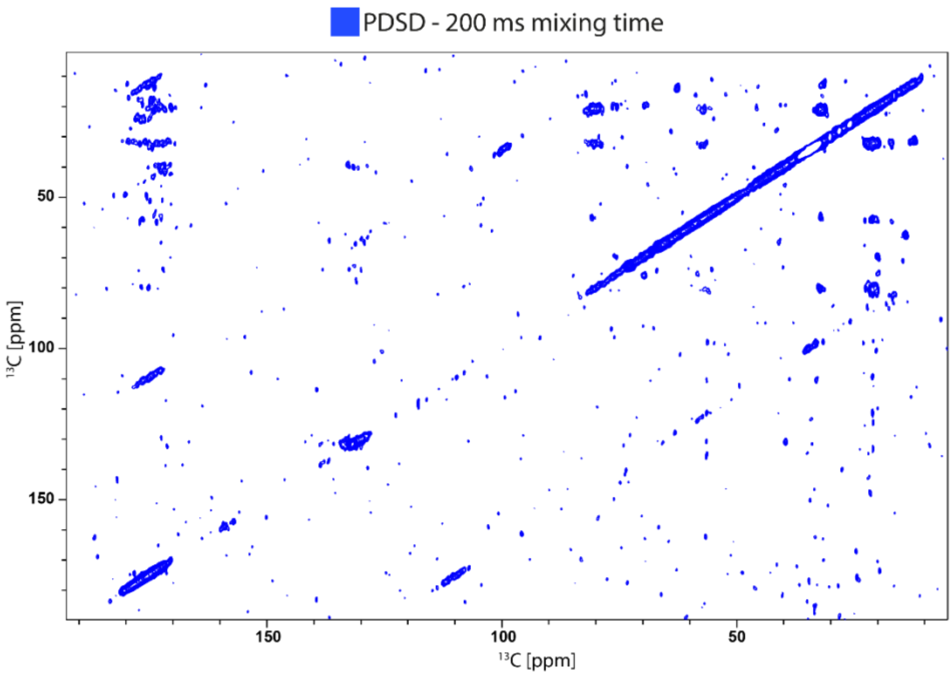

Figure S13: $^{13}C^{13}C$-PDSD spectrum of $^{13}C^{15}N$-labeled FR bound to the membrane-anchored G_q heterotrimer with 200 ms mixing time.

This journal is © The Royal Society of Chemistry 20xx

Please do not adjust margins

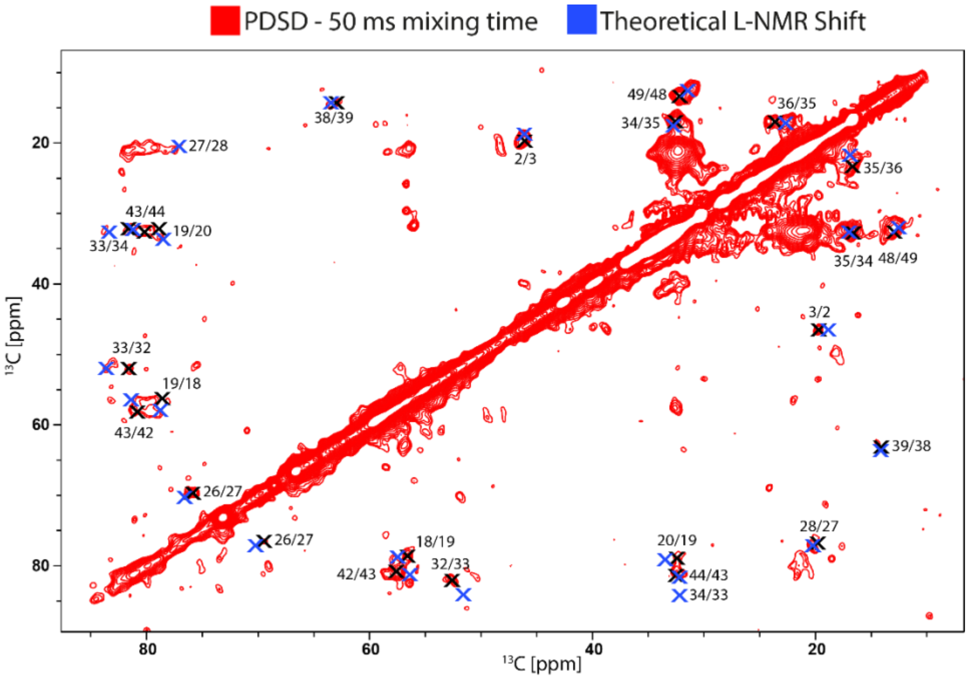

Figure S14: $^{13}C^{13}C$-PDSD spectrum of $^{13}C^{15}N$-labeled FR bound to the membrane-anchored G_q heterotrimer with 50 ms mixing time magnified to the Cα-Cβ region with annotated one-bond-correlation cross-peaks. The theoretical L-NMR chemical shift of the annotated signals is marked by blue crosses.

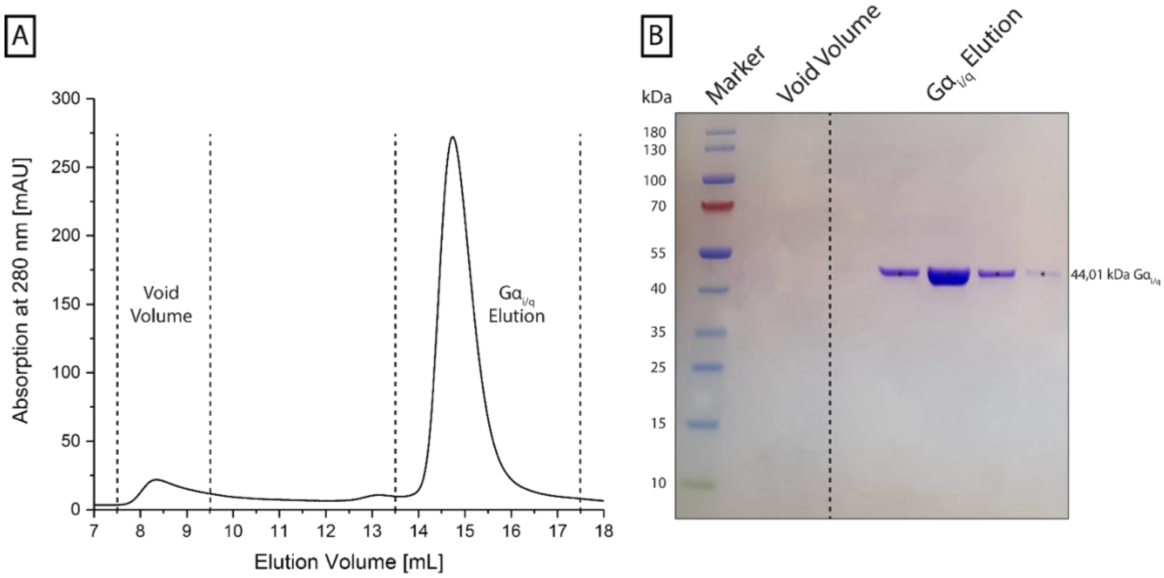

Figure S15: Analysis of $G\alpha_{i/q}$ protein expression. Size exclusion run of $G\alpha_{i/q}$ protein **(A)** and collected peak fractions analyzed by SDS-PAGE **(B)**. The band between 40 kDa and 55 kDa corresponds to $G\alpha_{i/q}$ (44,01 kDa).

Please do not adjust margins

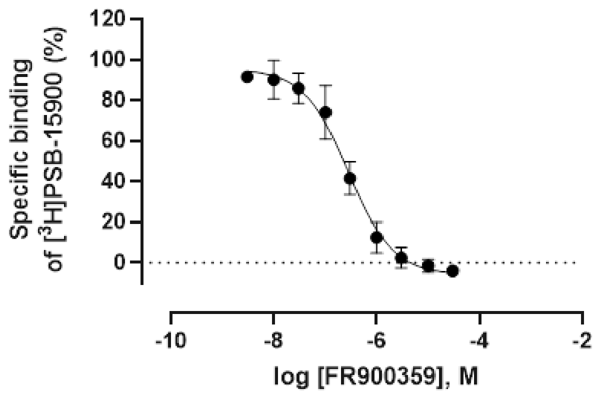

Figure S16: Radioligand binding assay using recombinant $G\alpha_{i/q}$ protein (5 µg) attached to metal resin beads via its 10x histidine tag. FR displaced [^{3}H]PSB-15900 (5 nM) binding in a concentration-dependent manner with an estimated K_D value of 0.271 ± 0.069 µM and a B_{max} value of 36.3 ± 8.8 pmol/mg of protein. These data demonstrate high affinity binding, although literature values for FR bound to G_q show even higher affinity of pK_i = 9.23.[2] The differences are due to chimeric and monomeric nature of $G\alpha_{i/q}$ used here. Data are means ± standard deviation from 3 separate experiments performed in duplicates.

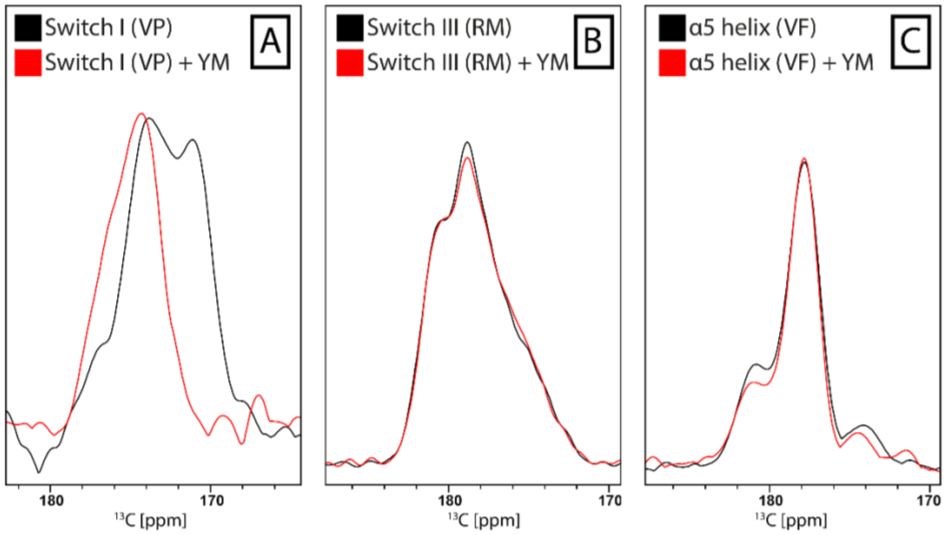

Figure S17: Spatially resolved protein response to YM binding by DNP-enhanced ssNMR. ^{13}C-NCOCX spectrum of frozen $G\alpha_{i/q}$ solution (black). Labeling schemes were chosen to detect one unique pair signal in switch I **(A)**, switch III **(B)** or in the α5 helix **(C)**. Addition of YM to the samples leads to narrowing of the signal in switch I, no effects in switch III and minor effects in the α5 helix (red).

This journal is © The Royal Society of Chemistry 20xx

Please do not adjust margins

(B) Tables

Table S1: Chemical shift assignment of $^{13}C^{15}N$-labeled FR in aqueous G_q protein buffer (L-NMR) and bound to reconstituted G_q protein in POPC:POPE:Cholesterol (5:4:1) proteoliposomes (ssNMR). [I/II/III] set of L-NMR resonances can be assigned to both atoms. [*] overlapping resonances.

Residue	Atom No.	Chemical Shift				
		L-NMR			ssNMR	
		^{1}H	^{13}C	^{15}N	^{13}C	^{15}N
Alanine	1	-	174	-	173,63	-
	2	5,25	46,48	-	46,11	-
	2-NH	7,39	-	118,17	-	118,77
	3	1,2	19,03	-	19,48	-
N-methyldehydroalanine	4	-	164,56	-	163,86	-
	5	-	143,76	-	N/A	-
	6a	6,02	125,72	-	N/A	-
	6b	3,6				
	7	2,95	39,77	-	40,53	-
	7-N	-	-	127,75	-	127,72
Phenyllactic acid	8	-	172,19	-	172,53	-
	9	5,74	73,36	-	73,07	-
	10a	3,28	40,71	-	40,12	-
	10b	3,19				
	11	-	N/A	-	N/A	-
	12/16	7,24	132.75	-	N/A	-
	13/15	7,23	131.25	-	N/A	-
	14	7,18	129.53	-	N/A	-
N-acetylhydroxyleucine	17	-	172,38	-	172,12	-
	18	5,07	56,58	-	56,68	-
	18-NH	8,85	-	116,53	-	116,95
	19	5,54	81,5	-	78,72	-
	20	1,9	32,41	-	32,24	-
	21[I]	0,88	21,37	-	21,04*	-
	22[I]	0,91	20,68	-	21,04*	-
	23	-	177,06	-	177,04	-
	24	2,14	24,79	-	24,47	-
N,O-dimethylthreonine	25	-	N/A	-	170,60	-
	26	3,84	70,2	-	69,71	-
	27	3,97	76,72	-	75,80	-
	28	1,33	20,26	-	20,00	-
	29	3,26	42,91	-	42,36	-
	29-N	-	-	114,86	-	114,98

Please do not adjust margins

	30	3,26	58.44	-	N/A	-
Hydroxyleucine	31	-	170,68	-	170,58	-
	32	5,27	52,21	-	52,01	-
	32-NH	7,03	-	107,36	-	107,66
	33	4,92	83,67	-	81,66	-
	34	2,16	32,67	-	32,49	-
	35[II]	1,01	17,07	-	16,86	-
	36[II]	0,95	22,93	-	24,03	-
N-methylalanine	37	-	174,53	-	174,92	-
	38	3,7	63,42	-	62,80	-
	39	1,43	14,52	-	14,04	-
	40	3,19	40,38	-	39,86	-
	40-N	-	-	118	-	118,30
N-propionylhydroxyleucine	41	-	177,22	-	176,23	-
	42	5,06	57,34	-	57,72	-
	42-NH	7,7	-	111,24	-	117,05
	43	4,01	79	-	80,95	-
	44	1,71	33,29	-	32,30	-
	45[III]	1	21,76	-	21,04*	-
	46[III]	0,94	21,73	-	21,04*	-
	47	-	178,49	-	179,79	-
	48	2,41	31,84	-	32,02	-
	49	1,2	12,59	-	13,09	-

Table S2: Parameters of spin relaxation (T_1, T_2), chemical exchange (k_{ex}) and ps-dynamics (hetNOE) of $^{13}C^{15}N$-FR in solution.

Residue	T_1 [ms]	T_2 [ms]	k_{ex} [s^{-1}]	hetNOE
**2-NH**	871 ± 9.5	58.4 ± 2.1	2700 ± 390	-1.74 ± 0.04
**18-NH**	880 ± 19.2	322 ± 3.0	735 ± 38	-1.56 ± 0.03
**32-NH**	805 ± 11.7	178 ± 6.4	1500 ± 179	-1.66 ± 0.03
**42-NH**	1080 ± 3.7	439 ± 9.3	736 ± 46	-1.67 ± 0.02

References

1. L. C. Ngoka and M. L. Gross, A nomenclature system for labeling cyclic peptide fragments, *J Am Soc Mass Spectrom*, 1999, **10**, 360-363.
2. J. H. Voss, M. Crusemann, C. R. O. Bartling, S. Kehraus, A. Inoue, G. M. Konig, K. Stromgaard and C. E. Muller, Structure-affinity and structure-residence time relationships of macrocyclic G alpha(q) protein inhibitors, *Iscience*, 2023, **26**.
3. K. Biemann, Mass spectrometry of peptides and proteins, *Annu Rev Biochem*, 1992, **61**, 977-1010.
4. P. Roepstorff and J. Fohlman, Proposal for a Common Nomenclature for Sequence Ions in Mass-Spectra of Peptides, *Biomed Mass Spectrom*, 1984, **11**, 601-601.

This journal is © The Royal Society of Chemistry 20xx

Please do not adjust margins
